# Coordination of mitochondrial and cellular dynamics by the actin-based motor Myo19

**DOI:** 10.1242/jcs.255844

**Published:** 2021-05-20

**Authors:** Katarzyna Majstrowicz, Ulrike Honnert, Petra Nikolaus, Vera Schwarz, Stefanie J. Oeding, Sandra A. Hemkemeyer, Martin Bähler

**Affiliations:** Institute of Molecular Cell Biology, Westfalian Wilhelms University Münster, 48149 Münster, Germany

**Keywords:** Cell adhesion, Mitochondria, Mitosis, Myosin

## Abstract

Myosin XIX (Myo19) is an actin-based motor that competes with adaptors of microtubule-based motors for binding to the outer mitochondrial transmembrane proteins Miro1 and Miro2 (collectively Miro, also known as RhoT1 and RhoT2, respectively). Here, we investigate which mitochondrial and cellular processes depend on the coordination of Myo19 and microtubule-based motor activities. To this end, we created Myo19-deficient HEK293T cells. Mitochondria in these cells were not properly fragmented at mitosis and were partitioned asymmetrically to daughter cells. Respiratory functions of mitochondria were impaired and ROS generation was enhanced. On a cellular level, cell proliferation, cytokinesis and cell–matrix adhesion were negatively affected. On a molecular level, Myo19 regulates focal adhesions in interphase, and mitochondrial fusion and mitochondrially associated levels of fission protein Drp1 and adaptor proteins dynactin and TRAK1 at prometaphase. These alterations were due to a disturbed coordination of Myo19 and microtubule-based motor activities by Miro.

## Abstract (precis)

**Summary:** An in-depth cellular and molecular characterization of myosin XIX (Myo19)-deficient cells reveals that different cellular and molecular processes are linked to mitochondrial dynamics regulated by Myo19.

## INTRODUCTION

Mitochondria are DNA-containing membrane-limited organelles that cannot form *de novo* and therefore, have to be partitioned during cell division between the two daughter cells. They can adopt different morphologies, for example, reticular networks, elongated tubules or individual short elements. A regulated morphological remodelling of the mitochondria is observed during the cell cycle. During G1/S phase, mitochondria form extended networks, whereas in mitosis they form short, fragmented organelles. This ensures their equal segregation during cytokinesis (Margineantu et al., 2002; Mandal et al., 2005; Schieke et al., 2008). The morphological dynamics of mitochondria is controlled by fusion and fission events. These produce the mixing and the segregation, respectively, of the mitochondrial DNA (mtDNA), proteins and lipids that are essential for mitochondrial and cellular homeostasis (Hoppins et al., 2007). Mitochondria contain two membranes, an outer and an inner membrane (OMM and IMM, respectively). The fusion of two mitochondria involves first the OMM before the IMM is able to fuse. Fusion of the OMM requires the mitofusins (Mfn1 and Mfn2) and IMM optic atrophy-1 protein (Opa1) (Cipolat et al., 2004; Koshiba et al., 2004; Meeusen et al., 2004). Similar to fusion, mitochondrial fission is a multistep process that involves the cooperation of various cellular components, such as tubules of the endoplasmic reticulum (ER), F-actin and the large dynamin-related GTPase protein Drp1 (also known as DNM1L) (Korobova et al., 2013, 2014; Moore et al., 2016). Its recruitment to mitochondria is mediated by several OMM proteins, including mitochondrial fission factor (Mff), mitochondrial fission protein 1 (Fis1), mitochondrial dynamics proteins (MiD49 and MiD51, also known as MIEF2 and MIEF1, respectively) and mitochondria-specific lipids (e.g. cardiolipin) (Loson et al., 2013; Macdonald et al., 2014). Post-translational modifications of Drp1 regulate its activity during the cell cycle. Phosphorylation of the serine residue at position 616 by two mitotic kinases increases its GTPase activity, while phosphorylation at Ser637 by cAMP-dependent protein kinase inhibits it (Chang and Blackstone, 2007; Taguchi et al., 2007; Kashatus et al., 2011). In interphase, Drp1 can be sumoylated and, during mitosis, de-sumoylated, which increases its activity (Harder et al., 2004).

Mitochondrial inheritance also depends on the proper subcellular distribution of mitochondria. Directed movement of mitochondria along polar microtubules and actin filaments is driven by force-generating motors. The bidirectional movement of mitochondria along microtubules is coordinated by the interaction with kinesins and dynein–dynactin complexes (Varadi, 2004; Pilling et al., 2006; Drerup et al., 2017; López-Doménech et al., 2018). In particular, anterograde, plus-end directed transport of mitochondria involves kinesin motors (Kif5 family) and retrograde (minus-end directed) transport involves dynein–dynactin. They are both recruited to the outer mitochondrial membrane proteins Miro1 and Miro2 (collectively Miro, also known as RhoT1 and RhoT2, respectively) by the adaptor trafficking kinesin proteins TRAK1 and TRAK2 (TRAK1/2; Milton in *Drosophila*) (Stowers et al., 2002; Brickley et al., 2005; van Spronsen et al., 2013; Gama et al., 2017). Miro has two GTPase domains that flank two consecutive Ca^2+^-binding EF hand pairs with ligand mimic helix arrangement (ELM domains). It is anchored in the OMM by a C-terminal transmembrane domain (Fransson et al., 2003; Klosowiak et al., 2013). Intriguingly, Miro was shown to be coupled to the IMM through the mitochondrial contact site and cristae organizing system (MICOS), which ensures force transduction of the motors to both membranes simultaneously (Modi et al., 2019). In addition to the transport of mitochondria, Miro proteins regulate mitochondrial morphology, ER–mitochondria contacts, mitophagy and cell migration (Misko et al., 2010; Kornmann et al., 2011; Kazlauskaite et al., 2014; Kanfer et al., 2015). Miro1 has also been shown to control lymphocyte and MEF adhesion through the regulation of mitochondrial distribution (Morlino et al., 2014; Schuler et al., 2017).

Transport along actin filaments is mediated by myosins. A class V myosin, Myo2, has been observed to control the mitochondria distribution and inheritance in budding yeast (Altmann et al., 2008). In *Drosophila* neurons, myosins V and VI have been shown to regulate mitochondrial length and antero- and retrograde transport (Pathak et al., 2010). Moreover, in mammalian cells myosin VI (Myo6) is involved in the formation of actin cages isolating damaged mitochondria in Parkin-mediated mitophagy (Kruppa et al., 2018). Cytoplasmic myosin II on the other hand contributes to mitochondrial fission (Korobova et al., 2014). The myosin Myo19 was found to associate with mitochondria and regulate their shape and movement (Quintero et al., 2009). Overexpression of Myo19 induced the formation of tadpole-shaped mitochondria (Quintero et al., 2009) and a perinuclear accumulation of mitochondria (Oeding et al., 2018). Downregulation of Myo19 compromised the proper partitioning of mitochondria during mitosis and induced a stochastic failure of cytokinesis (Rohn et al., 2014). *In vitro* studies of recombinant Myo19 showed that it is a plus-end-directed motor that stays attached to actin filaments for a large fraction of its chemo-mechanical cycle (Adikes et al., 2013). It consists of a head region, a neck region harbouring 3 IQ motifs and a tail region. The three IQ-motifs bind regulatory light chains (RLCs) of non-muscle and smooth muscle myosin 2 (Lu et al., 2014). The relatively short tail region (146 aa in human Myo19) is unique and responsible for binding to mitochondria through an interaction with lipids (Hawthorne et al., 2016; Shneyer et al., 2016) and Miro (López-Doménech et al., 2018; Oeding et al., 2018; Bocanegra et al., 2020). Interestingly, the dissociation of Myo19 from Miro leads to its degradation (López-Doménech et al., 2018; Oeding et al., 2018). Based on this fact, it could be shown *in vivo* that Myo19 competes with TRAK1/2, the adaptors for microtubule-based motors, for binding (Oeding et al., 2018). Thus, Miro coordinates actin- and microtubule-based movements.

To further characterize the coordination of actin- and microtubule-dependent mitochondria dynamics during different phases of the cell cycle and its importance for mitochondrial and cellular physiology, we created and characterized Myo19-deficient cells. In this process, we attempted to associate the observed phenotypes to specific activities of Myo19.

## RESULTS

### Full-length Myo19 is necessary for proper mitochondrial distribution during mitosis and cytokinesis

**Fig. 1.**
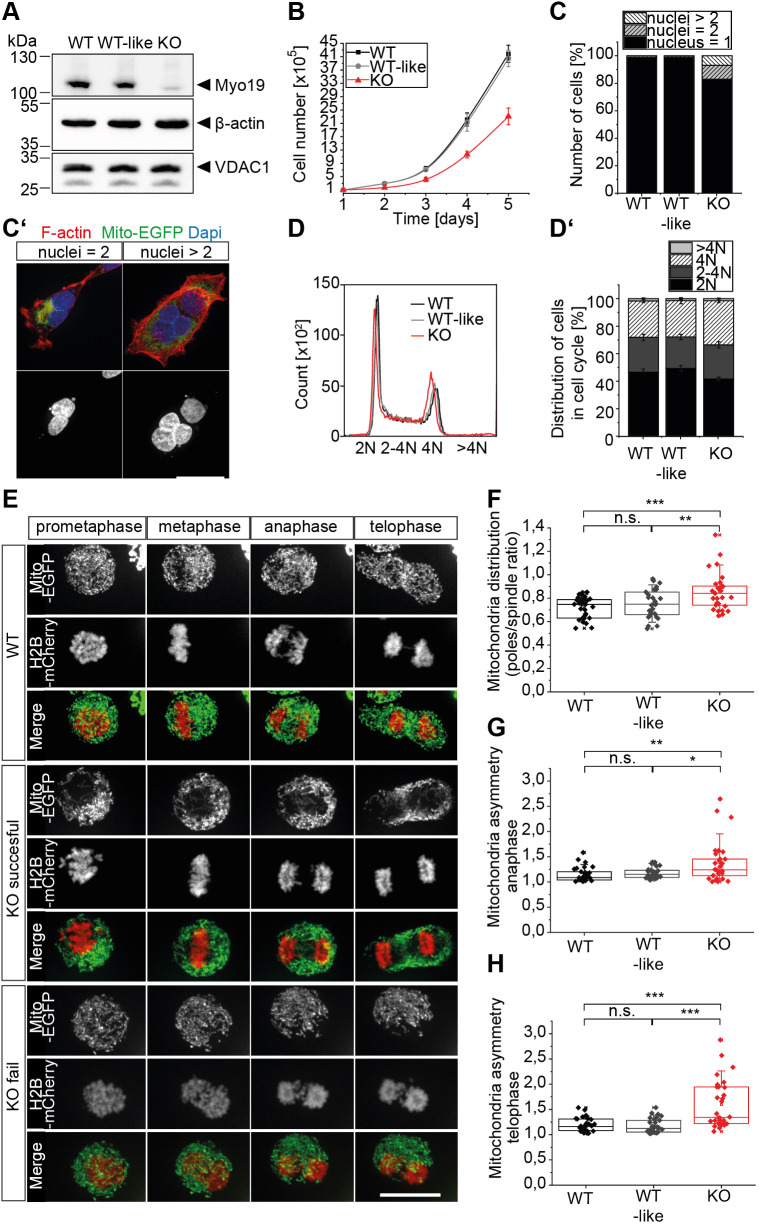
**Asymmetric partitioning of mitochondria during mitosis and stochastic cell division failure in Myo19-KO cells.** (A) Immunoblot confirming the knockout of Myo19 in HEK 293T cells. (B) Cell proliferation of different cell clones was analyzed over several days as indicated. *n*=7. (C) Quantification of the number of nuclei per cell in the indicated cell lines. Data are from three independent experiments with *n*≥176 cells analysed. (C′) Representative images of double and multinucleated (>2 nuclei) Myo19-KO cells. Cells were transfected with Mito-EGFP and stained with Texas Red–phalloidin and DAPI. Scale bar: 20 µm. (D,D′) Histograms and bar graphs of cell DNA content. Cells were stained with propidium iodide (PI) and analysed by flow cytometry for DNA content. Data are represented as mean± s.e.m. and are from seven independent experiments. (E) Frames from live-cell imaging of cells released from prometaphase (STLC block) showing mitochondria (Mito-EGFP, green) and chromosomes (H2B-mCherry, red). Scale bar: 20 µm. See also Movies 1–3. (F) Quantification of mitochondria enrichment at the cell poles at anaphase as determined by the ratio of mitochondria at poles to on the spindle (poles/spindle ratio). (G,H) Quantification of the asymmetrical mitochondria distribution in cells at anaphase and telophase. Data in F–H are displayed as box plots with the corresponding 75th and 25th percentile and the median. The whiskers show the 10th–90th percentiles. The minimum and maximum values are highlighted with dashes. Data are from *n*≥10 with at least 30 cells analysed per cell clone. ***P≤0.001; **P≤0.01; *P≤0.05; n.s., not significant (one-way ANOVA with Bonferroni post-hoc test). WT, wild-type; WT-like, wild-type-like; KO, Myo19-deficient cells.

Cells undergo extensive cellular reorganisation during mitotic entry and cytokinesis. To study the role of Myo19 in mitochondrial dynamics during those phases of the cell cycle, we created Myo19-knockout HEK 293T cell clones with CRISPR/Cas9 (Fig. 1A). We observed that Myo19-deficient (KO) cells were growing slower in comparison to wild-type (WT) or wild-type-like (WT-like) cells (control cells, unmodified by the CRISPR/Cas9 treatment) (Fig. 1B). This finding might be explained by a stochastic failure of cell division as has been reported for Myo19 knockdown cells (Rohn et al., 2014). Therefore, we counted the nuclei per cell using a blind test. We observed a significant increase (∼20%) of multinucleated cells in Myo19-KO clones in comparison to WT and WT-like cells (Fig. 1C,C′). Further analysis of DNA content by flow cytometry confirmed that an increased number of Myo19-deficient cells were tetraploid (Fig. 1D,D′).

Next, we analysed whether Myo19 regulates cellular organisation by controlling the actin and microtubule cytoskeleton or the distribution of mitochondria. We did not detect any differences in the organisation and structure of actin filaments or microtubules. Mitochondria were slightly less well spread within the cell (Fig. S1). To investigate whether Myo19 regulates the distribution of mitochondria during mitosis and cytokinesis, we synchronised the cells at prometaphase with the kinesin Eg5 inhibitor S-trityl-L-cysteine (STLC). After wash out of the drug, we monitored the cells by time-lapse imaging. Cells expressing Mito-EGFP and H2B-mCherry revealed that Myo19-KO cells frequently struggled to accomplish cytokinesis or failed to divide. Moreover, those cells often displayed changes in the morphology and distribution of mitochondria without abnormalities in chromosomes capture and segregation (Fig. 1E; Movies 1–3). We quantified the distribution of mitochondria at anaphase as a ratio of mitochondrial signal at cell poles versus the middle zone. During anaphase, mitochondria occupied mostly areas at the cell poles in an asymmetric manner in Myo19-KO cells, while in WT and WT-like cells mitochondria were localised more towards the cell equator (0.853±0.029; 0.718±0.017 and 0.750±0.022, respectively; all results in the main text are given as mean±s.e.m.) (Fig. 1F). Next, we analysed the segregation of the mitochondria to the two halves of mitotic cells separated by the equatorial plane at both anaphase and telophase. At anaphase, mitochondria were distributed asymmetrically in Myo19-KO cells (1.379±0.075), but not in WT (1.145±0.026) or WT-like cells (1.180±0.020) (Fig. 1G). The asymmetric distribution persisted during telophase in Myo19-KO cells (1.595±0.093), whereas in WT and WT-like cells mitochondria were rather distributed equally between the two forming daughter cells (1.196±0.026 and 1.173±0.028, respectively) (Fig. 1H). These results imply that Myo19 ensures an even inheritance of mitochondria during cell division. To analyse whether the lack of Myo19 affects specifically the even inheritance of mitochondria, we examined the distribution of the endoplasmic reticulum and of peroxisomes during mitosis and cytokinesis. In contrast to mitochondria, the distribution of these two organelles was not significantly altered at anaphase when Myo19 was missing (Fig. S2A–D). Interestingly, the distribution of the ER, but not of the peroxisomes was significantly asymmetric in Myo19-KO cells at telophase (1.356±0.062, *P*=0.015) (Fig. S2E). This finding implies a role for Myo19 and mitochondria in ER organisation during late stages of mitosis and cell division. No effect on peroxisome organisation could be detected.

**Fig. 2.**
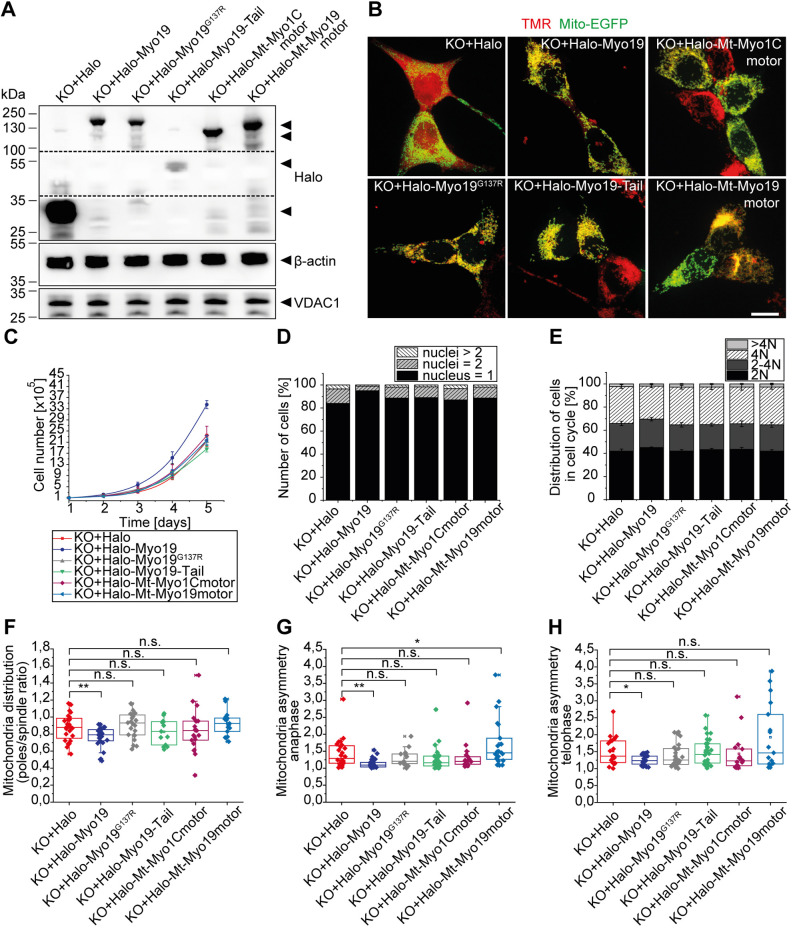
**Asymmetric partitioning of mitochondria during mitosis and stochastic failure of cytokinesis in Myo19-KO cells can be rescued by Myo19, but not by individual Myo19 domains.** (A) Immunoblot verifying the generation of Myo19-deficient cell lines stably expressing different Myo19 constructs as indicated on top. Immunoblots were developed with antibodies directed against Halo-Tag, β-actin and VDAC1, respectively. Arrowheads point at the respective constructs. The dashed lines reflect the borders of a membrane slice. (B) Cellular localization of Halo-tagged constructs. Cells were transfected with Mito-EGFP to label the mitochondria and incubated with TMR-ligand to label the indicated Halo-fusion constructs (red). Scale bar: 20 µm. (C) Cell proliferation was analysed for different cell clones as indicated; mean±s.e.m., *n*=4 independent experiments. (D) Quantification of the number of nuclei per cell in the different cell clones as indicated; *n*=3 independent experiments, *N*≥159 cells. (E) Quantification of DNA content by flow cytometry analysis of cells stained with propidium iodide (PI). Data are represented as mean±s.e.m. for 4 independent experiments. (F) Quantification of mitochondria distribution at cell poles versus spindle at anaphase for cells of the indicated cell lines. (G,H) Quantification of the asymmetric mitochondria distribution in cells at anaphase and telophase. Data in F–H are displayed as box plots with the sample median of at least 20 cells, together with the corresponding 75th and 25th percentiles. The whiskers show the 10th–90th percentiles. The minimum and maximum values are highlighted with dashes. *n*≥7. **P≤0.01; *P≤0.05; n.s., not significant (one-way ANOVA with Bonferroni post-hoc test and Mann–Whitney *U*-test).

To confirm the specificity of the Myo19-knockout phenotype, and to identify Myo19 activities that are able to abrogate the phenotype partially or completely, we performed rescue experiments in Myo19-KO cells. We created stable cell lines expressing either Halo-tag alone (KO+Halo) as a control or fused N-terminally to various Myo19 or Myo1C constructs. In addition to functional full-length Myo19 (KO+Halo-Myo19), we constructed a Myo19 motor mutant replacing glycine 137 with arginine (KO+Halo-Myo19^G137R^), which is predicted to abolish nucleotide binding (Bejsovec and Anderson, 1990) and a truncated Myo19 encompassing only the tail region (aa 824–970; KO+Halo-Tail). Furthermore, we artificially targeted the Myo19 and Myo1C motor regions to the outer mitochondrial membrane by replacing their tail regions with the transmembrane domain of Miro1 (KO+Halo-Mt-Myo19motor and KO+Halo-Mt-Myo1Cmotor, respectively) (Fig. S3). Myo19-KO cells were transfected with the individual constructs and stable cell lines were selected. The expression levels for all Halo constructs were comparable (Fig. 2A). To verify the mitochondrial localisation of the constructs, stable cells were transiently transfected with Mito-eGFP. The Halo tag fused to the constructs was labelled specifically with a tetramethylrhodamine (TMR) ligand. The TMR signal in Myo19-KO cells expressing Halo-tag alone was entirely cytosolic, whereas all Myo19 and Myo1C constructs localised to the mitochondria (Fig. 2B). The mitochondria-associated constructs did not appear to alter the steady-state mitochondria and actin filament organisation. Further analysis revealed that only recombinant expression of Halo-Myo19 was able to significantly increase Myo19^−/−^ cell proliferation in comparison to the KO+Halo cells (Fig. 2C). The number of cells with more than one nucleus and an increased DNA content were only reduced upon expression of full-length Myo19, but not with any of the other constructs. In recombinant Myo19-expressing cells, two or more nuclei were found in less than 4% of cells. In contrast, cells expressing any of the other constructs showed a higher number of multinucleated cells (∼15%) (Fig. 2D,E). Time-lapse imaging of dividing cells showed that in Halo-Myo19 expressing cells mitochondria were redistributed from the poles towards the cell centre (0.763±0.024) when compared to cells expressing Halo alone (0.879±0.032), Halo-Myo19^G137R^ motor mutant (0.905±0.033), Halo-Myo19-Tail (0.827±0.042), Halo-Mt-Myo1Cmotor (0.842±0.054) and Halo-Mt-Myo19motor construct (0.926±0.034) (Fig. 2F). The mitochondrial segregation at anaphase and telophase was largely asymmetrical in Myo19-KO cells expressing Halo-tag alone (anaphase 1.437±0.089; telophase 1.516±0.086), Halo-Myo19^G137R^ (1.308±0.073; 1.395±0.068), Halo-Myo19-Tail (1.288±0.082; 1.474±0.065), Halo-Mt-Myo1Cmotor (1.329±0.108; 1.434±0.139) and Halo-Mt-Myo19motor (1.728±0.156; 1.924±0.238). In contrast, in Halo-Myo19-expressing cells the distribution of mitochondria was rather uniform between the two halves of the cells at anaphase (1.137±0.027) and at telophase (1.217±0.027) (Fig. 2G,H). Together, these results indicate that only full-length functional Myo19 is able to prevent the abnormal accumulation of the mitochondria at the poles of mitotic cells and the unequal segregation of mitochondria.

### Coordination of mitochondria distribution during mitosis by Myo19 and microtubule-dependent motors

**Fig. 3.**
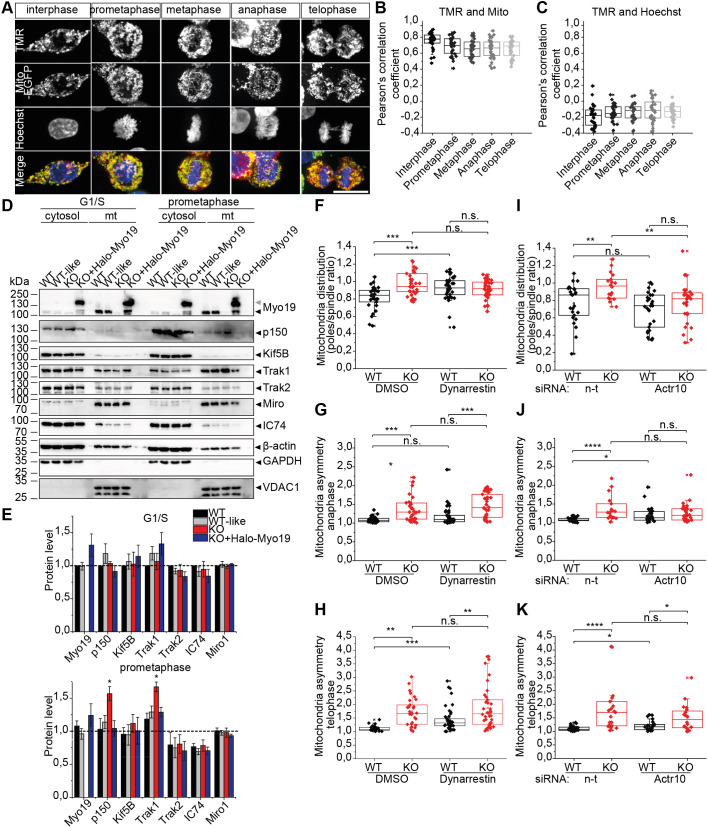
**Increased association of p150glued and Trak1 with mitochondria isolated from mitotic Myo19-KO cells and implications of inhibiting dynein.** (A) Representative images of cells in interphase and during mitosis stably expressing Halo-Myo19. Cells were transfected with Mito-EGFP and stained with TMR-ligand and Hoechst 34580. Scale bar: 20 µm. See also Movie 4. (B,C) Quantification of the correlation between the localization of the TMR-ligand (Halo-Myo19 construct), Mito-eGFP and Hoechst 34580. Myo19 remains associated with mitochondria at all phases of the cell cycle. (D) Representative immunoblots with indicated antibodies after cell fractionation into cytosol and mitochondria. Grey arrowhead indicates Halo-Myo19. (E) Quantification of proteins associated with purified mitochondria from synchronized cells in G1/S phase and prometaphase. Data are from 3–7 independent experiments; error bars represent ±s.e.m. *P≤0.05 (paired two-tailed Student's *t*-test). (F,I) Quantification of mitochondria distribution at cell poles versus the spindle at anaphase for cells of the indicated cell lines that were treated with DMSO or Dynarrestin and non-targeting (n-t) or Actr10 siRNA, respectively. (G,H,J,K) Quantification of the uneven mitochondrial distribution between the two halves of a cell at anaphase and telophase. Cells were treated with DMSO or Dynarrestin and n-t or Actr10 siRNA, respectively. Data are displayed as box plots with the corresponding 75th and 25th percentile. The whiskers show the 10th–90th percentiles. The minimum and maximum values are highlighted with dashes. *n*≥6 independent experiments, *N*≥30 cells. ****P≤0.0005; ***P≤0.001; **P≤0.01; *P≤0.05; n.s., not significant (one-way ANOVA with Bonferroni post-hoc test).

We subsequently examined, by confocal microscopy, whether Myo19 remains associated with mitochondria during mitosis. We transfected Myo19-KO cells stably expressing Halo-Myo19 with Mito-EGFP and stained them with Halo-tag TMR ligand and Hoechst 34580 (Fig. 3A; Movie 4). As shown previously, during interphase Myo19 colocalized with mitochondria, as demonstrated by the high value of the Pearson's correlation coefficient for TMR and Mito-eGFP (0.750±0.019). Myo19 TMR did not colocalize with the Hoechst 34580 signal (0.189±0.025). Cells that had entered mitosis still showed a colocalisation of Myo19 with mitochondria at prometaphase, although with a slightly reduced Pearson's correlation coefficient (0.687±0.021; *P*=0.02) and this colocalisation remained unaltered through the following mitotic stages (
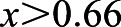
 for all division stages) (Fig. 3B,C). These results demonstrate that Myo19 remains attached to the mitochondria during all phases of the cell cycle.

Actin- and microtubule-based movements of mitochondria are coordinated by the shared receptor Miro. Therefore, the asymmetric inheritance of mitochondria and the stochastic failure of cell division by Myo19-null cells could be due to uncoordinated microtubule-based movement of the mitochondria. In support of this idea, forced attachment of overexpressed kinesin Kif5B and dynein to mitotic mitochondria leads to a similar phenotype to that we observed here in Myo19-KO cells (Chung et al., 2016). To determine whether the loss of Myo19 affected the association of the microtubule-based motor proteins and adaptors with mitochondria during mitosis, we synchronised the cells either by a double thymidine block in early S-phase or by STLC treatment in prometaphase. Synchronised cells were fractionated into cytosol and mitochondria (Fig. S4A). Western blot analysis showed no major differences in cytosolic protein levels (Fig. S4B) between different cell clones at interphase. Mitochondria purified from Myo19-KO cells at prometaphase, however, showed increased levels of bound dynactin subunit p150glued (also known as DCTN1) and TRAK1 (1.52- and 1.41-fold change in comparison to WT; *P*=0.041 and 0.030, respectively). The mitochondrially associated levels of these proteins could be restored to WT levels by the expression of full-length Halo-Myo19. Kinesin (Kif5B), dynein intermediate chain (IC74; also known as DYNC1I1) and Trak2 levels remained unchanged as the level of Miro (Fig. 3D,E). Of note, when Myo19 was missing the levels of adaptor proteins for microtubule-based motor proteins associated with purified mitochondria were increased exclusively during mitosis.

We further examined whether inhibition of dynein would be able to rescue the mitotic Myo19-KO phenotype. To do so, we either inhibited dynein acutely using 25 µM dynarrestin or through downregulation by means of siRNA against actin-related protein 10 (Actr10), a subunit of the dynein-associated dynactin complex. Actr10 downregulation at both interphase and prometaphase was confirmed by western blotting (Fig. S4D). Treatment of WT cells with dynarrestin at anaphase led to an enhanced localisation of mitochondria at the poles when compared to WT cells treated only with the solvent (0.923±0.022 versus 0.816±0.024). No further increase in poles:spindle ratio of the mitochondria was observed in Myo19-KO cells that had an increased localisation of the mitochondria at the poles even without any treatment (0.975±0.024 for DMSO control and 0.913±0.018 for inhibitor). Interestingly, downregulation of Actr10 in Myo19 KO cells rescued mitochondria distribution at anaphase and shifted it towards the cell equator, while in WT cells it did not significantly affect the ratio of mitochondria at poles to those at the spindle (0.961±0.035 and 0.778±0.044 with non-targeting RNA; 0.765±0.043 and 0.694±0.040 with Actr10 siRNA) (Fig. 3F,I). However, neither treatment with dynarrestin nor with Actr10 siRNA resulted in a rescue of the asymmetric partitioning of mitochondria at anaphase and telophase in Myo19-KO cells. Interestingly, dynarrestin treatment did not affect the asymmetry of mitochondria distribution at both anaphase and telophase in WT and Myo19 KO cells, except for an increase in asymmetric mitochondria distribution in WT cells at telophase (from 1.116±0.018 to 1.443±0.064) (Fig. 3G,H). In contrast, Actr10 siRNA treatment increased the asymmetry of mitochondria distribution in WT cells both at anaphase and telophase (from 1.085±0.011 to 1.208±0.042 at anaphase and from 1.105±0.017 to 1.213±0.042 at telophase), but did not affect it in Myo19 KO cells (from 1.377±0.076 to 1.265±0.047 at anaphase and from 1.840±0.205 to 1.507±0.110 at telophase) (Fig. 3J,K). In conclusion, to ensure proper mitochondria distribution during mitosis, Myo19 and dynein activities need to be tightly coordinated, as it is the case for kinesin and dynein activities.

### Myo19 regulates mitochondrial fragmentation at mitosis

**Fig. 4.**
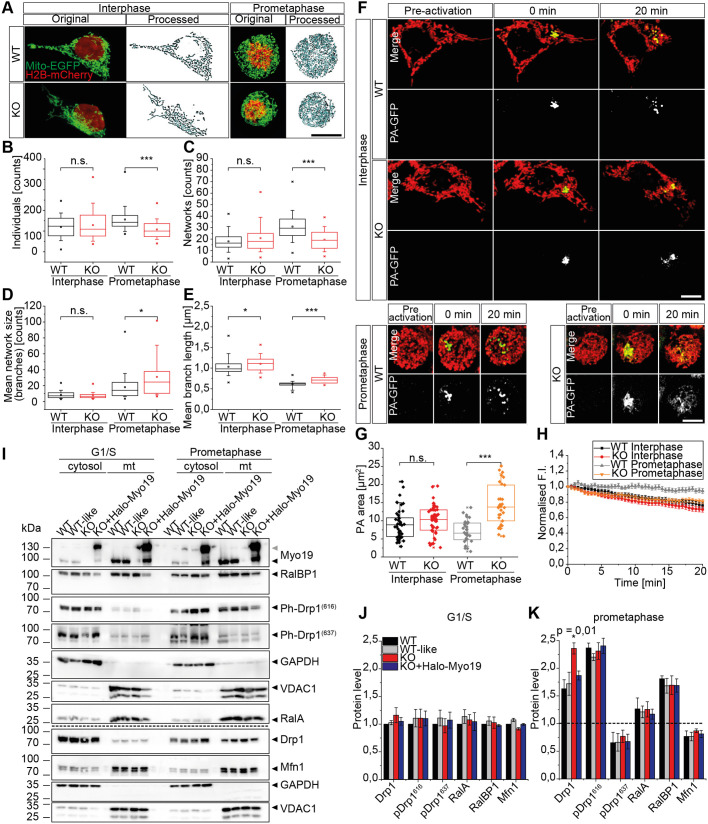
**Mitochondria in Myo19-KO cells are less fragmented at prometaphase.** (A) Representative images of wild-type (WT) and Myo19-KO (KO) cells expressing Mito-EGFP and H2B-mCherry at interphase (left) and prometaphase (right) with the corresponding processed and skeletonized mitochondrial networks used for analysis. Scale bar: 20 µm. (B–E) Mitochondrial network analysis as indicated on each graph performed for cells at interphase or arrested at prometaphase. Box plots show median (horizontal lines), boxes ranging from 25th to 75th percentiles, whiskers ranging from 10th–90th percentiles. *n*=3 independent experiments, *N*=90 cells. *P≤0.05; ***P≤0.001; n.s., not significant (Mann–Whitney *U*-test). (F) Live-cell imaging of WT and Myo19-KO cells (KO) synchronized at interphase (upper panel) and prometaphase (lower panel) expressing photoactivatable Mito-GFP (PA-GFP-Mito, green). Mitochondria were stained with Mitotracker Orange (red). Video frames shown were taken before, immediately after and 20 min. past photoactivation of a ROI. Scale bars: 10 µm. (G) Quantification of the area covered by photoactivated GFP 30 s after photoactivation. Data are displayed as box plots with the corresponding 75th and 25th percentile, the median and maximum values as dashes. *n*≥4 independent experiments, *N*≥30 cells. ***P≤0.001; n.s., not significant (Mann–Whitney *U*-test). (H) Fluorescence intensity (F.I.) PA-GFP-Mito probe diffusion over a 20-min period following photoactivation. See also Movies 5–8. (I) Immunoblots with indicated antibodies of cells at interphase and arrested at prometaphase fractionated into cytosol and mitochondria. Two different blots were assembled, as indicated by a dashed line. Grey arrowhead indicates Halo-Myo19. (J,K) Quantification of proteins associated with purified mitochondria from cells arrested at either G1/S phase (interphase) or prometaphase. Protein levels were normalized to VDAC1 and are presented as fold changes compared to WT at interphase. Data are from 3–7 independent experiments; error bars represent ±s.e.m. *P≤0.05 (paired two-tailed Student's *t*-test).

As cells enter mitosis, their mitochondria undergo extensive fragmentation. However, mitochondria in cells that lacked Myo19 appeared to be less fragmented during mitosis. To study the mitochondrial morphology, we arrested cells expressing Mito-EGFP and H2B-mCherry either in interphase or prometaphase, and analysed the mitochondrial morphology using total projections of *Z*-stacks and the semi-automated MiNa plug-in (Valente et al., 2017) (Fig. 4A). We did not observe major changes in mitochondrial morphology in Myo19-KO cells synchronised at interphase except for the mean branch length, which was slightly increased in Myo19-KO cells compared to WT cells (1.033±0.031 μm and 1.103±0.025 μm, respectively; *P*=0.017) (Fig. 4B–E). However, mitochondria in Myo19-KO cells clearly exhibited an altered morphology at prometaphase when compared to mitochondria of WT cells. The number of mitochondrial individuals (156±9 in WT and 107±8 in KO) and networks (WT 31±2 and KO 20±2) was decreased, indicating that the mitochondria were more elongated (Fig. 4B,C). At prometaphase, mitochondria in Myo19-KO cells additionally formed a highly interconnected network, as revealed by more pronounced branching. The number of branches was increased from 18±2.5 in WT cells to 31±4.5 in Myo19 KO cells and the branch length was also increased from 0.613±0.014 μm in WT cells to 0.873±0.166 μm in Myo19 KO cells (Fig. 4D,E). Mitochondrial morphology and dynamics at interphase and at prometaphase stage of the cell cycle was further investigated using photo-activatable GFP targeted to the mitochondrial matrix (PA-GFP-Mito). In cells, PA-GFP-Mito was activated in a defined region of interest (ROI; 2.56 µm×2.56 µm) and development of the fluorescence was monitored by live-cell imaging. In cells at interphase there were no significant differences in the area covered by activated PA-GFP-Mito between WT and Myo19-KO cells (9.07±0.612 μm^2^, 10.21±0.563 μm^2^, respectively; *P*=0.098). However, in agreement with the morphological analysis of a more interconnected network in Myo19-KO cells at prometaphase compared to WT cells, the fluorescence spread after photoactivation over a significantly larger area in Myo19 KO cells than in WT cells (14.430±1.021 μm^2^, 7.056±0.548 μm^2^, respectively). Moreover, we measured the spread of PA-GFP-Mito across the mitochondrial population by quantifying its dilution. At interphase, the fluorescence decay, resulting from the diffusion within the mitochondrial network, mitochondrial fusion, and mitochondrial movement, was comparable between WT and Myo19-KO cells. However, in Myo19-KO cells synchronised at prometaphase, the mitochondria labelled by photoactivation were fusing with unlabelled mitochondria causing a reduction in fluorescence intensity. In contrast, in WT cells mitochondria did not fuse with each other and fluorescence intensity (MFI) remained constant (Fig. 4F–H; Movies 5–8).

Based on these results, the increased connectivity of mitochondria in Myo19 KO cells at prometaphase appears to be due to a lack of downregulation of mitochondrial fusion during mitosis. However, this does not exclude a simultaneously impaired rate of fission. In interphase cells, mitochondria fission is regulated by the ER, which induces mitochondria constriction sites (Friedman et al., 2011). Therefore, we examined ER organisation in Myo19-KO cells during mitosis. The analysis of the ER (RFP-KDEL) structure did not reveal any major differences between WT and Myo19-KO cells, but as already mentioned, ER distribution was altered at telophase in cells lacking Myo19 (Fig. S2B).

To address how Myo19 regulates mitochondrial interconnectivity specifically in mitosis, we performed subcellular fractionation of cells synchronized in interphase and prometaphase, respectively, and monitored the association of fission- and fusion-related proteins with mitochondria (Fig. 4I). There were no alterations in protein levels observed in cytosol and mitochondria fractions from different cell clones arrested in interphase (Fig. 4J; Fig. S4C). Levels of proteins involved in mitochondrial fusion, namely Mfn1 and Opa1, were not altered in mitochondria purified from Myo19-KO cells (Fig. 4I–K; Fig. S4E). Intriguingly, we observed that significantly higher Drp1 protein levels were associated with mitochondria isolated from prometaphase Myo19-KO cells than WT cells (1.446-fold change, *P*=0.010). Expression of recombinant Myo19 restored WT Drp1 protein levels associated with mitochondria purified from cells arrested at prometaphase (Fig. 4J,K). Drp1 activity can be either activated or inhibited by phosphorylation. No differences in Drp1 phosphorylation were detected between Myo19-KO and WT cells. Consistent with this, the levels of RalA or RalBP1, proteins that mediate Drp1 activation and relocation to mitochondria (Kashatus et al., 2011), were not altered in the mitochondrial fraction isolated from Myo19-KO cells (Fig. 4J,K). Collectively, these results indicate that during mitosis Myo19 regulates the amount of DRP1 that is associated with mitochondria.

### Myo19 deficient cells display lower respiratory capacity

**Fig. 5.**
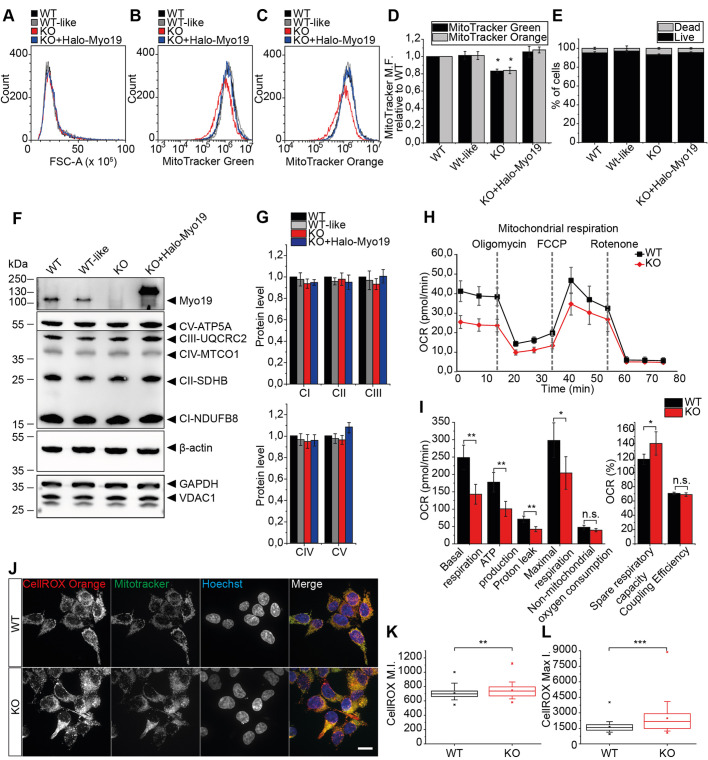
**Myo19-KO cells display lower respiratory capacity and higher ROS generation.** (A–C) Wild-type (WT), WT-like, Myo19-KO (KO) and Myo19-KO expressing Halo-Myo19 (KO+Halo-Myo19) cells were labelled with 50 μM Mitotracker Green (MTG) or 50 μM Mitotracker Orange (MTO) together with ZOMBIE-NIR and analysed by flow cytometry. Histograms of cells analysed for cell size (FSC-A), MTO and MTG fluorescence are shown. (D) Quantification of mean fluorescence (M.F.) intensity of MTG and MTO dye normalised to FSC-A. (E) Histograms of the percentage of live and dead cells measured based on ZOMBIE-NIR MFI. Data were compared to WT. *n*=7 for Mitotracker staining and *n*=3 for ZOMBIE-NIR staining. Data are represented as mean±s.e.m. *P≤0.05 (one-way ANOVA and Mann–Whitney *U*-test). (F,G) Representative immunoblot of OXPHOS complexes in indicated cell lines and quantification. (H,I) Cells were analysed with a Mito stress test from Seahorse technology. Mitochondria respiration profiles of WT and KO cells are shown after indicated drug injections. (I) Quantification of measurements displayed as oxygen consumption rate (OCR). Data are represented as mean±s.e.m. from three independent experiments with each cell line represented in five wells per experiment. **P≤0.01; *P≤0.05; n.s., not significant (Mann–Whitney *U*-test). (J) Representative images of WT and Myo19-KO cells stained with CellROX Orange to measure oxidative stress in live cells, with Mitotracker Green (MTG) and Hoechst 34580 to visualize mitochondria and nuclei, respectively. (K,L) Quantification of mean (M.I) and maximum (Max I.) fluorescence intensity of CellROX per cell. Data are displayed as box plots with the median, the corresponding 75th and 25th percentiles. The whiskers show the 10th–90th percentiles. The minimum and maximum values are highlighted with dashes. *n*=4 independent experiments, *N*=120 cells. ***P≤0.001; **P≤0.01 (Mann–Whitney *U*-test).

Altered dynamics can disturb mitochondrial potential, result in the accumulation of oxidized proteins and overall affect cell respiration (Twig et al., 2008). We analysed mitochondrial mass and potential in Myo19-deficient cells by flow cytometry using Mitotracker Green (MTG) and Mitotracker Orange (MTO) dyes, respectively. Cells lacking Myo19 showed a reduction in both MTG and MTO mean fluorescence intensity by 17%, but no differences in cell size. Mitotracker mean fluorescence was restored to WT levels in KO cells when recombinant Myo19 was expressed (Fig. 5A–D). The differences in MTG and MTO fluorescence intensities between the various cell clones were not due to increased cell death, as ZOMBIE-NIR dye staining showed comparable amounts of live and dead cells (Fig. 5E). Reduced MTG and MTO fluorescence intensities in Myo19-deficient cells could indicate either a reduced mitochondria mass or an altered mitochondria morphology and protein organisation. There appeared to be no differences in mitochondrial mass, as we could not detect any significant differences in VDAC1 levels or the levels of various OXPHOS complexes between the cell clones (Fig. 5F,G).

Furthermore, to assess whether Myo19 KO was affecting mitochondrial activity, we measured the oxygen consumption rate (OCR) using the Mito Stress Test from Seahorse XF technology. This enables a detailed analysis of mitochondrial function by the inhibition of selected OXPHOS steps using different drug treatments. Addition of oligomycin inhibits ATP-linked respiration by blocking ATP synthase. The remaining basal respiration can be attributed to proton leakage. Furthermore, addition of FCCP ionophore leads to an inner membrane potential collapse and a rapid oxygen consumption to maximum levels. The addition of rotenone allows the contribution of OXPHOS to total respiration to be assessed, as it inhibits the mitochondrial complex I. The difference between maximal and basal respiration indicates the spare capacity of mitochondrial OXPHOS and can be taken as a marker of cell fitness and adaptability. The remaining oxygen consumption is of non-mitochondrial origin. The proportion of the ATP-linked respiration to the basal respiration describes the coupling efficiency. The respiration profile revealed reduced mitochondria activity in Myo19-KO cells in comparison to WT cells (Fig. 5H). Myo19-KO cells showed a reduction by 40% in the oxygen demand under baseline conditions (basal respiration), ATP production and proton leak (*P*≤0.002). The addition of FCCP led to a rapid oxygen consumption in both cell lines, though Myo19 KO cells reached 30% lower maximal rates (*P*=0.03). Interestingly, Myo19 KO cells showed a 15% increase in spare respiratory capacity in comparison to WT cells (*P*=0.02), with no differences in coupling efficiency nor in non-mitochondrial oxygen consumption (Fig. 5I). Taken together, these results show an altered mitochondrial respiration for Myo19-KO cells.

Oxygen consumption, ATP production and reactive oxygen species (ROS) generation can serve as indicators of mitochondrial health and function. Mitochondria are targets and sources of oxidants and oxidative stress was shown to affect Mitotracker mean fluorescence (Doherty and Perl, 2017). Therefore, we sought to investigate the ROS levels in Myo19-deficient cells using the ROS indicator dye CellROX Orange (Fig. 5J). Myo19-KO cells showed an increase in mean (5%) and maximum (40%) CellROX fluorescence intensity when compared to the WT cells (*P*=0.029 and *P*=5.449×10^−4^, respectively) (Fig. 5K,L). The increased ROS levels in Myo19-KO cells could explain the altered mitochondrial functionality and further exacerbate ROS production.

### Loss of Myo19 leads to reduced cell adhesion

**Fig. 6.**
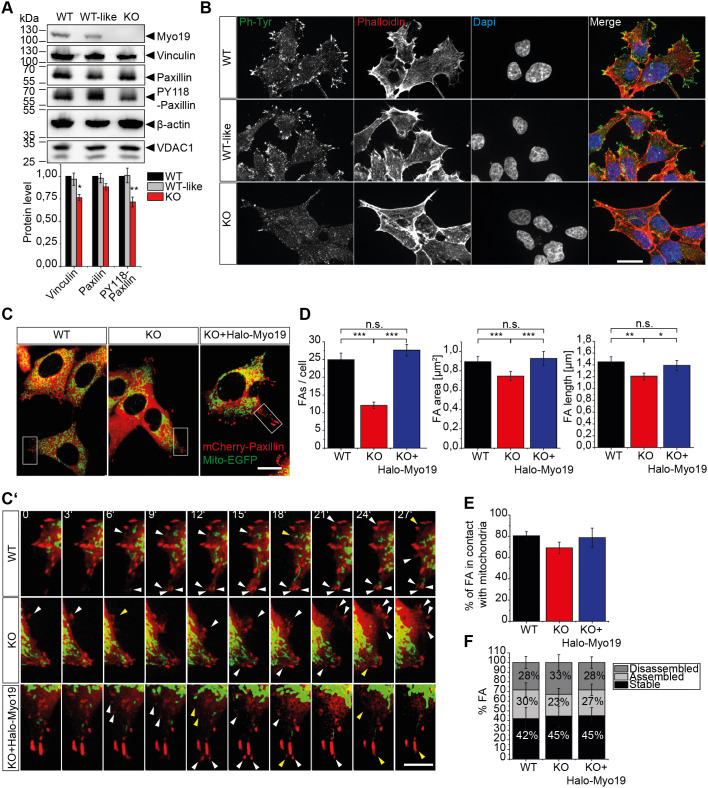
**Myo19-KO cells demonstrate a reduced cell-matrix adhesion.** (A) Immunoblots and quantification of focal adhesion proteins as indicated in WT, WT-like and Myo19-deficient cells. Data were normalized to β-actin and compared to WT cells. Data are represented as mean±s.e.m. and are from 5–8 independent experiments. **P≤0.01; *P≤0.05 (paired two-tailed Student's *t*-test). (B) Representative images of wild-type (WT), wild-type-like (WT-like) and Myo19 knockout (KO) cells stained with phospho-tyrosine (Ph-Tyr) antibodies to visualize focal adhesions. Actin filaments were labelled with phalloidin and nuclei with DAPI. Scale bar: 20 µm. (C,C′) WT, Myo19-KO and Myo19-KO expressing Halo-Myo19 cells were transiently transfected with mCherry-Paxillin and Mito-EGFP and imaged for 30 min with 1 min intervals by confocal microscopy. C′ shows an enlargement of the boxed region in C. White arrowheads indicate assembly and yellow arrowheads disassembly of focal adhesions (FAs). (D) Quantification of FA number and morphology as indicated. Data are represented as mean±s.e.m. *n*=6 independent experiments with at least 60 cells analysed. ***P≤0.001; **P≤0.01; *P≤0.05; n.s., not significant (one-way ANOVA with Bonferroni post-hoc test). (E) Quantification of mitochondria and focal adhesion contacts. Data are represented as mean±s.e.m. *n*=6 independent experiments with at least 60 cells analysed. (F) Focal adhesion complexes that remained stable, disassembled or newly assembled were quantified per cell over 30 min. *n*=6 independent experiments, *N*=30 cells. All data show mean±s.e.m. See also Movies 9–13.

**Fig. 7.**
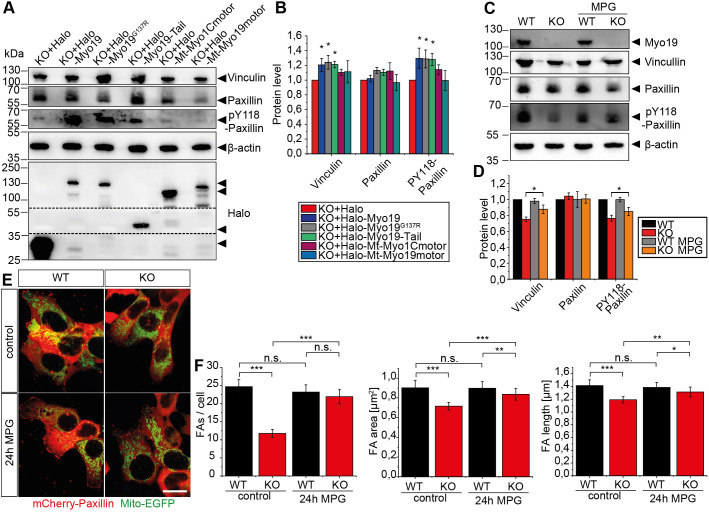
**Alterations in Myo19-KO cell focal adhesion number and shape can be rescued by the tail region of Myo19 and the quenching of ROS.** (A) Immunoblot of protein levels in Myo19-KO cell clones stably expressing different Halo constructs as indicated. The dashed lines reflect the borders of a membrane slice. (B) Quantification of focal adhesion protein levels. The band intensities were normalized to β-actin and fold changes of proteins were compared to KO cells expressing Halo alone. Data are represented as mean±s.e.m. from *n*=4 independent experiments. *P≤0.05 (paired two-tailed Student's *t*-test). (C) Immunoblot of protein levels in untreated and MPG-treated cells as indicated. (D) Quantification of focal adhesion (FA) protein levels with band intensities normalized to β-actin and fold changes of proteins compared to WT cells. Data are represented as mean±s.e.m. from 4 independent experiments. *P≤0.05 (paired two-tailed Student's *t*-test). (E) WT and Myo19-KO cells were transiently transfected with mCherry-Paxillin and Mito-EGFP, and incubated for 24 h in control medium or medium supplemented with 1 mM MPG. (F) Quantification of FA number and morphology as indicated. Data are represented as mean±s.e.m. *n*=3 with at least 90 cells analysed. ***P≤0.001; **P≤0.01; *P≤0.05; n.s., not significant (one-way ANOVA with Bonferroni post-hoc test).

We noticed that Myo19-depleted cells were detaching from the surface more often than WT cells. This prompted us to take a look at the levels of proteins participating in cell adhesion. We found that in Myo19-KO cells the level of phosphorylated paxillin (PY118) was significantly reduced (28%; *P*=0.004), with total levels of paxillin unchanged (Fig. 6A). Moreover, levels of vinculin were decreased (23.7% less; *P*=0.0156) in Myo19-KO cells in comparison to the WT cells (Fig. 6A). Immunofluorescence staining of focal adhesions with phospho-tyrosine antibodies revealed that Myo19-KO cells have less prominent focal adhesions (Fig. 6B). To further investigate focal adhesion dynamics, we co-transfected cells with mCherry-Paxillin and Mito-EGFP to visualise focal adhesions and mitochondria simultaneously (Fig. 6C,C′). In comparison to WT cells, Myo19-KO cells displayed reduced numbers of focal adhesions per cell (25±2; 12±1, respectively), with a smaller area (0.893±0.056 μm^2^ and 0.746±0.042 μm^2^, respectively) and length (1.431±0.069 μm^2^; 1.192±0.055 μm, respectively). Expression of recombinant Myo19 rescued focal adhesion number, average focal adhesion area and length compared to control cells (27±2; 0.928±0.079 μm^2^; 1.395±0.085 μm, respectively) (Fig. 6D). When we looked at the interplay between mitochondria and focal adhesions, we did not observe significant differences in mitochondria–focal adhesion encounters (*P*=0.318) (Fig. 6E). Furthermore, the dynamics of focal adhesions was not significantly different between WT and KO cells (Fig. 6F; Movies 9–13). Since expression of fully functional Halo-Myo19 was able to rescue this phenotype, we further examined whether the expression of the different Myo19 constructs could restore the adhesion protein levels. Interestingly, levels of vinculin and phosphorylated paxillin were elevated in Myo19-KO cells expressing any of the Myo19 tail region-containing constructs, such as Halo-Myo19, Halo-Myo19^G137R^ motor mutant and Halo-Myo19-Tail. Constructs that did not contain the tail region such as the Halo-tag or mitochondrially targeted Myo1C and Myo19 motor constructs did not rescue the levels of vinculin and phospho-paxillin (Fig. 7A,B). These results indicate that the changes in cell adhesion properties could involve Myo19 binding to Miro and its coordination of microtubule-based movements of mitochondria.

A growing body of evidence indicates that ROS play major roles in intracellular signalling affecting cell adhesion. Given the enhanced ROS generation in Myo19-KO cells, we sought to investigate whether the focal adhesion phenotype could be rescued by the reduction of ROS. Interestingly, treatment of Myo19-KO cells with the free radical scavenger *N*-(2-mercaptopropionyl)glycine (MPG) led to higher levels of vinculin and phosphorylated paxillin in comparison to untreated cells (10.8% and 12% increase; *P*=0.046, *P*=0.044, respectively; Fig. 7C,D). Moreover, we observed an increased number of focal adhesions per cell in comparison to untreated cells (21±2). Focal adhesion area and length were also significantly increased in cells treated with ROS scavenger (0.837±0.059 μm^2^ and 1.313±0.077 μm^2^, respectively) (Fig. 7E,F). Thus, this data suggests that enhanced ROS production in Myo19-KO cells affects cell–matrix adhesion.

## DISCUSSION

Mitochondria distribution and dynamics are prerequisites for various cellular functions and cell homeostasis. Here, we characterized the functional implications of a loss of Myo19, an actin-based molecular motor that is stabilized by the binding to the outer mitochondrial membrane protein Miro. The loss of Myo19 affected during mitosis mitochondrial morphology and segregation and during interphase bioenergetics and cell adhesion (Table 1).

**Table 1.**
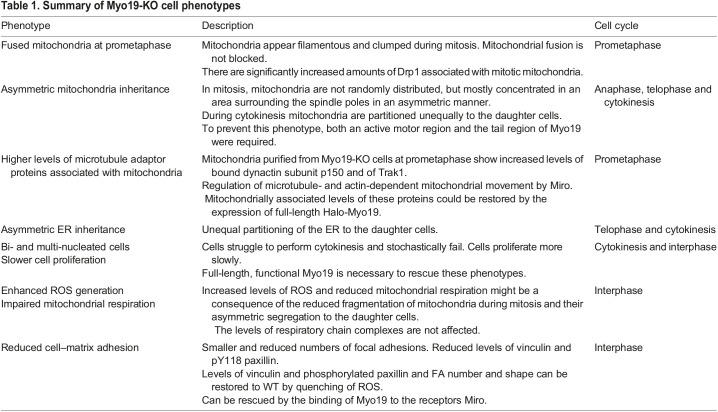
Summary of Myo19-KO cell phenotypes

Mitochondrial segregation and inheritance during cell proliferation has been reported to occur both passively and actively. Passive segregation is envisioned to rely on mitochondrial fragmentation followed by a release of the mitochondria from the cytoskeleton (Chung et al., 2016). On the other hand, highly ordered and selective segregation has been reported to depend on movement along microtubules and actin filaments (Lawrence and Mandato, 2013; Kanfer et al., 2015; Katajisto et al., 2015). The acute downregulation of Myo19 by siRNA in HeLa cells has been shown to result in a stochastic failure of cytokinesis and an asymmetric distribution of mitochondria during mitosis (Rohn et al., 2014). We confirm and extend these findings with HEK293T cells that exhibit a permanent total loss of Myo19 expression. These cells showed a decreased cell proliferation and were partially multinucleated due to a stochastic failure in cytokinesis. In mitosis, mitochondria were less fragmented and not randomly distributed, but instead situated mostly in an area at the spindle poles in an asymmetric manner. This gave rise to an unequal partitioning of the mitochondria to the daughter cells. To prevent this phenotype, it was not sufficient to direct the actin filament-interacting motor domains of either Myo19 or Myo1C to the mitochondria. Although we cannot exclude that a small non-mitochondrial cytosolic pool of Myo19 might contribute to the observed phenotypes, the results indicate that both an active motor region and the tail region of Myo19 are required for even segregation of mitochondria during cell division. Myo19 force production along actin filaments and force production along microtubules coordinated by the Myo19 tail region through competition for binding to Miro seems to be required for proper mitochondrial segregation at cell division. Thus, the coordination between actin- and microtubule-dependent motors by Miro appears to play a crucial role in mitochondria organisation during nuclear and cellular division. In agreement with this assumption, acute inhibition of dynein by dynarrestin or downregulation of the dynactin subunit Actr10 (Arp11) affected mitochondria distribution in mitosis, indicating that a fine balance of both actin-based Myo19 and microtubule-based dynein and kinesin activities is essential.

Mitochondria undergo defined shape changes during the cell cycle. In G1 phase mitochondria adopt a highly connected tubular morphology. The transition into S phase leads to mitochondrial hyperfusion allowing for a higher ATP output (Mitra et al., 2009). As the cells enter mitosis, mitochondria undergo extensive Drp1-dependent fragmentation (Taguchi et al., 2007). However, in Myo19-deficient cells mitochondria appeared to be filamentous and clumped during mitosis. Nevertheless, there were significantly increased amounts of Drp1 associated with mitotic mitochondria, potentially as a response to the continuing fusion of mitochondria in Myo19-deficient cells at prometaphase, whereas mitochondrial fusion paused in WT cells. Miro, the mitochondrial receptor for Myo19, not only affects mitochondria motility, but additionally morphology that is balanced by fusion and fission (Fransson et al., 2006; Saotome et al., 2008). Indeed, Miro has been shown to suppress Drp1-mediated fission of mitochondria and peroxisomes (Saotome et al., 2008; Covill-Cooke et al., 2020). Epidermal wounding in *C. elegans* triggers Miro-dependent, but Drp1-independent, mitochondrial fragmentation, which in turn causes an upregulation of mtROS (Fu et al., 2020). Furthermore, dynein–dynactin has been shown to regulate mitochondrial morphology by controlling the recruitment of Drp1 to the mitochondria (Varadi, 2004). We suggest that coordination of Myo19 and microtubule-based motors by Miro impacts mitochondrial fusion and fission through Drp1. This balance seems to be especially fine-tuned during mitosis.

The loss of Myo19 caused a stochastic failure of cytokinesis. In a previous Myo19 knockdown study, this phenotype could be rescued by decreasing mitochondrial fusion and it could be mimicked by inhibiting mitochondrial fission (Rohn et al., 2014). Therefore, cytokinesis might be blocked by an extensive mitochondrial network that cannot be partitioned properly.

Mitochondrial fusion–fission dynamics is coupled reciprocally with mitochondrial function. Inhibiting mitochondrial fission leads to a loss of mitochondrial DNA, a decrease of mitochondrial respiration and increased levels of reactive oxygen species (ROS) (Parone et al., 2008). Conversely, mitochondria with an increased membrane potential (ΔΨ_m_) have a higher, while those with a decreased potential have a lower, probability to fuse (Twig et al., 2008; Liu et al., 2009; Mishra and Chan, 2016). Intracellularly generated ROS can regulate mitochondrial shape and distribution (Debattisti et al., 2017). The mitochondrial respiration and ATP production in Myo19-deficient cells were reduced, although the levels of respiratory chain complexes were not decreased. Furthermore, Myo19-deficient cells also showed increased levels of ROS generation. These functional impairments of the mitochondria in Myo19-deficient cells might be a consequence of the reduced fragmentation of mitochondria during mitosis and their asymmetric segregation to the daughter cells. These two changes might both have a lasting effect well into interphase. Altered ROS and ATP levels or ATP:ADP ratios could contribute to the observed phenotypes of slowed cell proliferation and impaired matrix adhesion. Myo19-deficient cells had smaller and reduced numbers of focal adhesions. Indeed, there is ample precedence for an interdependence of mitochondria and focal adhesions. Mitochondria positioning has been shown to influence the dynamics of focal adhesion complexes (Mao et al., 2018). Adhesion in a human primary glioblastoma cell line was positively correlated with the cellular content of mitochondria (Caino et al., 2015) and targeted sequencing identified Myo19 as a familial glioma candidate gene (Jalali et al., 2015). It has been shown that mitochondria infiltrate the leading edge of migrating cells and regulate focal adhesion size (Daniel et al., 2019 preprint). Moreover, the rise in intracellular ROS levels was found to coincide with the detachment of epithelial and endothelial cells from the surface (Li et al., 1999). ROS have also been shown to modulate the activity of phosphatases and kinases and thus, influence cell adhesion properties (Li et al., 1999; Schieber and Chandel, 2014). Indeed, quenching of ROS in Myo19-deficient cells leads to a recovery of focal adhesion numbers and morphology. Integrin-linked kinase (ILK), when located at focal adhesions, blocked the interaction of Miro1 with TRAK2, which induced a perinuclear accumulation of mitochondria and a detrimental ROS generation (Onodera et al., 2018). We previously reported that Myo19 binds directly to Miro (Oeding et al., 2018). Recent studies have shown that Miro1 controls adhesion of lymphocytes and MEFs (Morlino et al., 2014; Schuler et al., 2017). Additionally, Miro1 and Miro2 double-knockout cells have been reported to frequently detach from the substrate during mitosis (López-Doménech et al., 2018). Thus, mitochondria positioning along actin filaments by Myo19 or along microtubules by kinesin and dynein could play a role in formation of focal adhesion complexes in the cell by affecting ROS generation. Reduced mitochondrial activity in Myo19-depleted cells could also influence focal adhesion dynamics, since the inhibition of mitochondrial ATP generation was shown to reduce focal adhesion number (Daniel et al., 2019 preprint). The analysis of focal adhesion proteins in Myo19-KO cells showed a significant reduction in vinculin and phosphorylated paxillin (PY118) levels. Paxillin mutations were shown to play a role in mitochondrial dynamics and influence lung cancer progression (Kawada et al., 2013). The Myo19 focal adhesion phenotype could be rescued by the expression of full-length Myo19, Myo19 motor mutant and the Myo19 tail. These results suggest that the changes in adhesion properties involve the binding of Myo19 to the receptors Miro. The competition for binding to Miro between the Myo19 tail and microtubule-based motors regulates the generation of ROS that in turn regulates cell adhesion.

In summary, the coordination by Miro of the actin-based motor Myo19 and of microtubule-based motors critically regulates mitochondria dynamics, inheritance and function. Furthermore, on a cellular level Myo19 regulates cell proliferation and adhesion.

## MATERIALS AND METHODS

### Construction of plasmids

Plasmids obtained from Addgene include: pmTagBFP2-TOMM20 N-10 (#55328), pEGFP-Peroxisome (#54501) and pmCherry-Peroxisome (#54520). Plasmids pmRFP-KDEL and pmCherry-H2B were gifts from Mario Schelhaas (Center for Molecular Biology of Inflammation, Münster), plasmid pmCherry-Paxillin was a gift from Anna Chrostek-Grashoff (Institute of Molecular Cell Biology, Münster) and plasmid pmRFPrubyLifeAct was a gift from Roland Wedlich-Söldner (Institute of Cell Dynamics and Imaging, Münster). The plasmid pMito-EGFP was constructed by inserting the mitochondrial targeting sequence of the subunit VIII of human cytochrome *c* oxidase from Red FP vector-Mitochondrion (BD Pharmingen) via NdeI/Bsh T1 into the pEGFP-C1 vector (Clontech). To construct the plasmid pMito-PAGFP, the sequence coding for the mitochondrial targeting sequence was excised and inserted into the vector pPAGFP-C1 (Patterson and Lippincott-Schwartz, 2002). The mitochondrial targeting sequence was further inserted into pmTagBFP2-N1, derived from pmTagBFP2-TOMM20-N-10 (Subach et al., 2011) to construct pMito-mTagBFP2. Human Myo19 sequences were inserted into the plasmid pIREShyg-Halo encompassing a multiple cloning site that was modified to harbour additional restriction sites. The plasmid pIREShyg-Halo was constructed by insertion of the HaloTag sequence that was amplified by PCR into plasmid pIREShyg. Subsequently, the sequence coding for full-length human Myo19 (Oeding et al., 2018) was inserted to get pIREShyg-Halo-Myo19. The point mutation coding for Myo19 G137R was introduced by QuikChange mutagenesis. The plasmid pIREShyg-Halo-Myo19 tail codes for amino acid residues 824–970 of Myo19 and was amplified by PCR for subcloning. The plasmid pIREShyg-Halo-Myo19head 3IQ-Miro1TM encompasses sequences coding for amino acid residues 1–830 of Myo19 fused to the transmembrane region of human Miro1 encoding residues 589–618. The sequence coding for rat Myo1c (myr 2) amino acid residues 1–769 was fused to the transmembrane region of Miro1 (residues 598–618) and inserted into pIREShyg-Halo yielding pIREShyg-Halo-Myo1c-head-3IQ-Miro1TM.

### Cell culture and generation of stable cell lines

HEK 293T cells (ECACC) were grown in Dulbecco's modified Eagle's medium (DMEM; P04-03550, PAN Biotech) supplemented with 100 U/ml penicillin; 100 µg/ml streptomycin (P06-07100, PAN Biotech) and 10% (v/v) heat inactivated FCS (Biochrom) (complete DMEM medium), at 37°C, 5% CO_2_ and 95% humidity. Cells were passaged 1:10 twice a week at 80–90% confluence. To generate the stable HEK Halo-tagged cell lines, 100,000 cells were seeded in a 6-well plate and 24 h later cells were transfected with 2.5 µg of the linearized plasmid DNA mixed with Lipofectamine Plus transfection reagents (15338100, Invitrogen) according to the manufacturer's instructions. Two days later cells were seeded in 150 mm dishes in complete DMEM medium supplemented with 100 µg/ml hygromycin (1358GR005, BioFroxx). Every third day, selective medium was changed. Cells viable after 2 weeks were considered as stably transfected. Individual colonies of cells were transferred to a 6-well plate for further expansion.

### CRISPR/Cas9 mediated gene knockout of human Myo19

The Myo19 knockout cell line was generated using CRISPR/Cas9-mediated genome editing as previously described (Cong et al., 2013). Guide RNAs (gRNAs) targeting exon 4 (5′-ACCTGGAAGAGATAACGTGAAGG-3′, 5′-GGCCACAATCCGGGGTCTGATGG-3′, 5′-CCCCACCCAGGAACTCCTGCAGG-3′, 5′-ACAAACTGGATGACCTCACCAGG-3′) of the *myo19* locus were designed for ‘quadruple nicking’ and cloned into two pX335B plasmids (gift from Dr Boris Greber) that contain two gRNA cassettes, D10A Cas9 nickase and a GFP-T2A-puromycin cassette. Transfection of both pX335B vectors was performed using Lipofectamine Plus transfection reagents (15338100, Invitrogen) according to the manufacturer's protocol. Individual EGFP-positive cells were sorted using a FACSAria (BD Bioscience) and clones were further propagated. Genomic modifications were verified by PCR (primers F1, 5′-CTGTTTTGAGTACCAGCTGTCG-3′; F2, 5′-CAAGCCAGGGAGTACCTCAGAG-3′; R, 5′-CCTGGAGAAGCTGTGCTGACTAC-3′, biomers.net GmbH) and western blot.

### Reagents and antibodies

The following monoclonal and polyclonal antibodies were used: anti-Actr10 [western blotting (WB): dilution 1:100, sc-515293, Santa Cruz Biotechnology], anti-β-actin (WB: 1 µg/ml, AC-15/A1978, Sigma-Aldrich), anti-β-tubulin [immunofluorescence (IF): dilution 1:200, T4026, Sigma-Aldrich], anti-Drp1 (WB: 1:1000, 5391S, Cell Signaling], anti-Drp1Ser616 (WB: dilution 1:1000, 3455, Cell Signaling), anti-Drp1Ser637 (WB: dilution 1:1000, 4867, Cell Signaling), anti-Dynein IC74 (WB: dilution 1:500, MAB1618, Millipore), anti-GAPDH (WB: 0.5 µg/ml, TA802519, OriGene), anti-HaloTag^®^ (WB: 1 µg/m, G9211, Promega), anti-Kif5B (WB: 0.141 µg/ml, ab167429, Abcam), anti-Miro1/2 (WB: 0.25 µg/ml, NBP1-59021, Novus Biologicals), anti-Mitofusin1 (WB: dilution 1:1000, ab57602, Abcam), anti-Myo19 (WB: 0.176 µg/ml, ab174286, Abcam), anti-OXPHOS (WB: dilution 1:1000, ab110413, Abcam), anti-p150glued (WB: 0.5 µg/ml, 610474, BD Transduction Laboratories), anti-paxillin (WB: 2 µg/ml, MA5-13356, Invitrogen), anti-Phospho-Paxillin (PY118, WB: 1 µg/ml, 44-722G, Thermo Fisher Scientific), anti-phospho-tyrosine (IF: 1:100, P3300, Sigma-Aldrich), anti-RalA (WB: 0.25 µg/ml, 610221, BD Transduction Laboratories), anti-RalBP1 (WB: 0.1 µg/ml, H00010928-M02, Novus Biologicals), anti-Trak1 (WB: 0.5 µg/ml, PA5-44180, Thermo Fisher Scientific), anti-Trak2 (WB: dilution 1:500, 13770-1-AP, Proteintech), anti-VDAC1 (WB: 1 µg/ml, ab14734, Abcam), anti-vinculin (WB: dilution 1:1000, V9131, Sigma-Aldrich), anti-mouse IgG-HRP (Goat, WB: dilution 1:5000, 115-035-003, Jackson ImmunoResearch), anti-rabbit IgG-HRP (goat, WB: dilution 1:5000, 111-035-003, Jackson ImmunoResearch) and anti-mouse-IgG-Alexa Fluor 488 (goat, IF: 1:500, 115-545-003, Jackson ImmunoResearch). The following additional reagents were used for the staining of cells: CellROX Orange dye (C10443, Thermo Fisher Scientific), FITC–phalloidin (IF: dilution 1:100, P5182, Sigma-Aldrich), HaloTag TMR Ligand (5 µM, G8251, Promega), HaloTag R110Direct (5 µM, G3221, Promega), Mitotracker Green FM (50 nM, M7514, Thermo Fisher Scientific), Mitotracker Orange CMXRos (50 nM, M7510, Thermo Fisher Scientific) and Texas-Red–phalloidin (IF: 1:100, T7471, Thermo Fisher Scientific).

### Western blotting

Cell homogenates were prepared in ice-cold NP-40 lysis buffer [50 mM Tris-HCl, pH 7.4, 10% (v/v) Glycerol; 100 mM NaCl, 2 mM MgCl_2_, 1% (v/v) NP-40, freshly added: 1 mM DTT, 10 µg/ml aprotinin, 10 µg/ml leupeptin, 10 µg/ml Pefabloc], boiled in SDS loading buffer and separated by SDS-PAGE. Proteins were transferred overnight on to PVDF membrane (03010040001, Roche, Sigma-Aldrich). The membrane was blocked in 5% non-fat dry milk in TBS with 0.05% Tween 20 (TBST) for 1 h at room temperature (RT) and incubated with primary antibodies overnight at 4°C. The membrane was washed three times with TBST (each wash 5 min) and incubated with the corresponding HRP-conjugated secondary antibody for 1 h at RT. After three washes with TBST the protein bands were detected with Super Signal West Pico Substrate (34078, Thermo Fisher Scientific) according to the manufacturer's protocol, using a ChemiDoc MP Imaging System (Bio-Rad).

### Cell proliferation assay

To monitor cell proliferation, 100,000 cells per well were seeded in 6-well plates and incubated for one to five days at 37°C, 5% CO_2_ and 95% humidity. Cells were stained with Trypan Blue and counted daily at approximately the same time using a counting chamber.

### Cell synchronisation

Cells were synchronised at prometaphase by treatment with 5 µM S-trityl-L-cysteine (STLC; 164739, Sigma) for 20 h. For biochemistry, cells were synchronized using 2 mM thymidine (#296542A, Santa Cruz Biotechnology) for 16 h and released into fresh complete medium for 8 h, followed by either 16 h of 2 mM thymidine (interphase block) or 20 h of 5 µM STLC (prometaphase block) incubation.

### Mitochondria purification

High-purity mitochondria were isolated using Qproteome Mitochondria Isolation Kit (QIAGEN) according to the manufacturer's protocol.

### Flow cytometry

Flow cytometry analysis was performed with a CytoFLEX instrument (B3-R2-V0, Beckman Coulter) using the CytExpert software (Beckman Coulter). Before each use, the calibration with CytoFLEX Daily QC Fluorospheres (B253230, Beckman Coulter) was executed and experiments were carried out using the manufacturer's sheath fluid (B51503, Beckman Coulter).

#### DNA content analysis

Cells (10^6^) were collected and fixed in ice-cold 70% ethanol for at least 2 h at −20°C. Next, ethanol was removed by centrifugation (twice at 700 ***g*** and once at 2800 ***g***, each 5 min, 4°C) and cells were resuspended in 500 µl of FxCycleTM PI/RNAse Solution (F10797, Life Technologies Thermo Fisher Scientific), gently vortexed and incubated for 30 min in the dark at RT. Cells were analysed using the PE channel from CytExpert software.

#### Analysis of mitochondrial mass and membrane potential

Adherent cells at a density of 1×10^6^ cells per dish were stained for 30 min at 37°C, 95% humidity and 5% CO_2_ either with 50 nM Mitotracker Green FM or 50 nM Mitotracker Orange CMXRos diluted in serum-free DMEM. Subsequently, cells were harvested and stained for 15 min at RT in the dark with 100 µl of ZOMBIE-NIR Dye (423105, BioLegend) diluted in 1× PBS 1:100. Cells were washed with cold 1× PBS with 2% FCS and analysed in 500 µl of 1× PBS with 2% FCS using FITC, PE and APC-A750 channels.

### Oxygen consumption measurements

The oxygen consumption rate (OCR) was measured using an XF24 Extracellular Flux Analyzer (Seahorse Bioscience). The sensor cartridge was calibrated overnight with 200 µl of XF Calibrant per well at 37°C. The assay plates were double coated with 20 µg/ml fibronectin (F2006, Sigma-Aldrich) and 0.1 mg/ml poly-L-lysine (P6282, Sigma-Aldrich), and 1×10^5^ cells per well were plated in complete DMEM medium in a 96-well XF Cell Culture Microplate 4 h before the analysis. Next, cells were incubated for 1 h in XFBase medium supplemented with 4.5 g/l glucose, 2 mM glutamine and 1 mM pyruvate. The assay was run in five replicates using the XF Extracellular Flux Analyzer (Seahorse Bioscience). Oxygen consumption was measured after injections of 1 µM oligomycin (port A), 0.5 µM FCCP (port B) and 0.5 µM rotenone (port C). The test parameters were generated automatically and calculated by the XF Mito Stress Test Report Generator (Seahorse Bioscience).

### Cell transfection and staining

#### Transfection

Cells were seeded on 20 µg/ml fibronectin coated (F2006, Sigma-Aldrich) coverslips in 24-well plates at a density of 20,000 cells per coverslip or in a 35 mm Ibidi µ-dish at a density of 40,000 cells. Transfection was performed using Lipofectamine Plus transfection reagents (15338100, Invitrogen). Briefly, 0.25 µl of Plus reagent was mixed with 0.25 µg of plasmid DNA and 1 µl of LTX in 50 µl DMEM, incubated for 5 min at RT and added dropwise onto cells. After 4 h of incubation the transfection mix was removed by washing twice with fresh complete medium. After 24 h of incubation, cells were examined by live-cell imaging, fixed or harvested for further analysis.

#### Staining

Cells were incubated with 50 nM Mitotracker Orange CMXRos diluted in growth medium for 15 min under normal growth conditions and then washed 3 times with fresh growth medium. Subsequently, coverslips were briefly washed with warm 1xPBS (137 mM NaCl, 2.7 mM KCl, 10.2 mM Na_2_HPO_4_, 1.8 mM KH_2_PO4, pH 7.0) and fixed with 4% paraformaldehyde in 1× PBS for 15 min at 37°C. Free aldehydes were blocked in 0.1 M glycine for 10 min and cells were mounted with Mowiol (3.4 mM Mowiol 4-88, DABCO). To stain cells for endogenous phospho-Tyr or β-tubulin, cells were permeabilized with 0.1% Triton X-100 in 1× PBS for 15 min and blocked with 5% normal goat serum (blocking buffer: 5% NGS in 1× PBS) for 1 h at RT. The primary antibody was diluted in blocking buffer and incubated with cells overnight at 4°C. Afterwards, cells were washed 3 times, 5 min each, and incubated with corresponding secondary antibody diluted in blocking buffer for 1 h at RT. Subsequently, cells were washed and mounted with Mowiol.

#### F-actin staining

Fixed and permeabilized cells were blocked in 5% NGS for 15 min and then stained with the dye-coupled phalloidin diluted in 1× PBS for 1 h at RT and mounted in Mowiol.

#### Halo-tag staining

Prior to fixation cells were stained for 15 min with 5 µM of either HaloTag^®^ TMR, R110Direct or Oregon Green ligand diluted in culture medium. Free TMR or Oregon Green ligands were removed by several washings, 5 min each, in warm medium and once with complete DMEM for 30 min. R110Direct stained cells were directly imaged.

#### ROS staining

Cells were first stained with 75 nM Mitotracker Green FM in complete DMEM at 37°C for 10 min, washed with warm 1× PBS and stained for 15 min with 5 µM Hoechst 34580. Immediately afterwards cells were stained with 5 µM CellROX Orange dye for 30 min under normal growth conditions. Cells were washed 3 times with complete imaging medium and directly analysed.

### Image acquisition and analysis

Images were acquired using a 63×/1.4 NA objective mounted on an UltraVIEW VoX spinning disk confocal laser-scanning microscope operated with Volocity software (PerkinElmer). Z-stacks across the depth of the cell were acquired for each experiment.

#### Mitochondria morphology analysis

Cells 24 h after transfection were synchronised in interphase or into prometaphase and imaged at increments of 0.1 µm in the *Z*-direction. The mitochondria morphology was assessed with the semi-automated MiNA toolset (Valente et al., 2017). Total projections of images were pre-processed by applying mask, CLAHE and top hat filter to enhance mitochondria structures. Binarized images were converted to skeleton structures, which were analysed and grouped into descriptive mitochondrial network parameters.

#### Live-cell imaging and analysis of mitosis and cell division

*Z*-stacks acquired with 1 μm increments and covering the entire depth of the transfected cells were taken in 6 min intervals. Stacks were compiled for analysis of total pixel intensity and ImageJ was used to assess mitochondria localisation. Background was subtracted and the summed mean fluorescence intensity (MFI) at the cell poles was divided by the MFI from the area in between to obtain the poles:spindle ratio. Values lower than 1 would indicate an accumulation of the organelles at the cell equator. Organelle symmetry at anaphase and telophase was measured by dividing the cell into two polygons and the MFI value of the larger area was divided by the MFI value of the smaller area. A ratio higher than 1 would indicate asymmetry.

#### Live-cell imaging and analysis of focal adhesion dynamics

Cells were imaged at 24 h post transfection in 1 min intervals for 30 min. For experiments with ROS scavenger, cells after transfection were incubated for 24 h in medium supplemented with 1 mM MPG (M6635, Sigma). Analysis of focal adhesion properties was performed using ImageJ as described in Horzum et al. (2014). Briefly, total projections of *Z*-stacks (1 μm steps) were processed by subtracting the background (rolling ball with 20 pixels radius), enhancing local contrast (CLAHE; 19 block size; 256 histogram bins; 5 maximum slope) and mathematical exponential (EXP). Brightness and contrast were adjusted automatically and Laplacian of Gaussian plug-in (Log3D; *X*=3; Y=3) was applied. In addition, a threshold was set so that ‘Analyze Particles’ command recognised focal adhesion. Focal adhesion and mitochondria contacts were assessed manually by tracking and scoring interactions over imaging time (the researcher was not blind to conditions). Focal adhesion dynamics, including assembly, disassembly and stability was counted and classified according to the existence in some or all of the time frames.

#### Photoactivation

At 24 h after cell transfection a ROI with 2.56×2.56 μm^2^ was selected for photoactivation of PA-GFP-Mito with a 405 nm laser (25% power). A sequence of images was collected from the cell bottom to the top in 1 μm steps (Z series) with 30 s intervals in the red (561 nm; 10% power, ex: 50 ms) and green (488 nm; 20%, ex: 50 ms) channels. Cells were imaged for 3 min before and 20 min after photoactivation. The images were thresholded and area of photoactivation was measured. The time-dependent (between *t*-0 and *t*-20 min post-photoactivation) decay in fluorescence intensity was measured using ImageJ Fiji software as described by Gomes et al. (2011).

#### Quantification of organelle distribution

The distribution of actin and mitochondria was measured as previously described (Kapitein and Hoogenraad, 2011) with some modifications. Sum projections of *Z*-stacks (increment 0.5 μm) were made binary. The distance of each pixel to the manually defined cell centre was calculated automatically. A plot of frequency versus distance was created. For statistical analysis the distance from the cell center at 95% of total organelle fluorescence was measured and plotted.

### Statistical analysis

Data are displayed as box-and-whisker plots. The boxes are 25th−75th quartiles, whiskers range from 10th to 90th percentiles, the mean is marked as a square and the median as a line. Bar graphs show mean±s.e.m. values. Using OriginPro 2015 SR2 (OriginLab Corporation), paired two-tailed Student's *t*-test was used to calculate the statistical significance against control values, one-way ANOVA with post-hoc Bonferroni test and Mann–Whitney *U*-test were performed for multiple and pairwise comparisons. Significance values are indicated as *P≤0.05; **P≤0.01; ***P≤0.001 and ****P≤0.0001.

## Supplementary Material

Supplementary information

Reviewer comments
