# Supplementary material for: Coordination of mitochondrial and cellular dynamics by the actin-based motor Myo19
Source: J Cell Sci. 2021 May 20;134(10):jcs255844. doi: 10.1242/jcs.255844 (PMC8186483; doi:10.1242/jcs.255844)
Supplement: Supplementary information [file joces-134-255844-s1.pdf]

Fig. S1

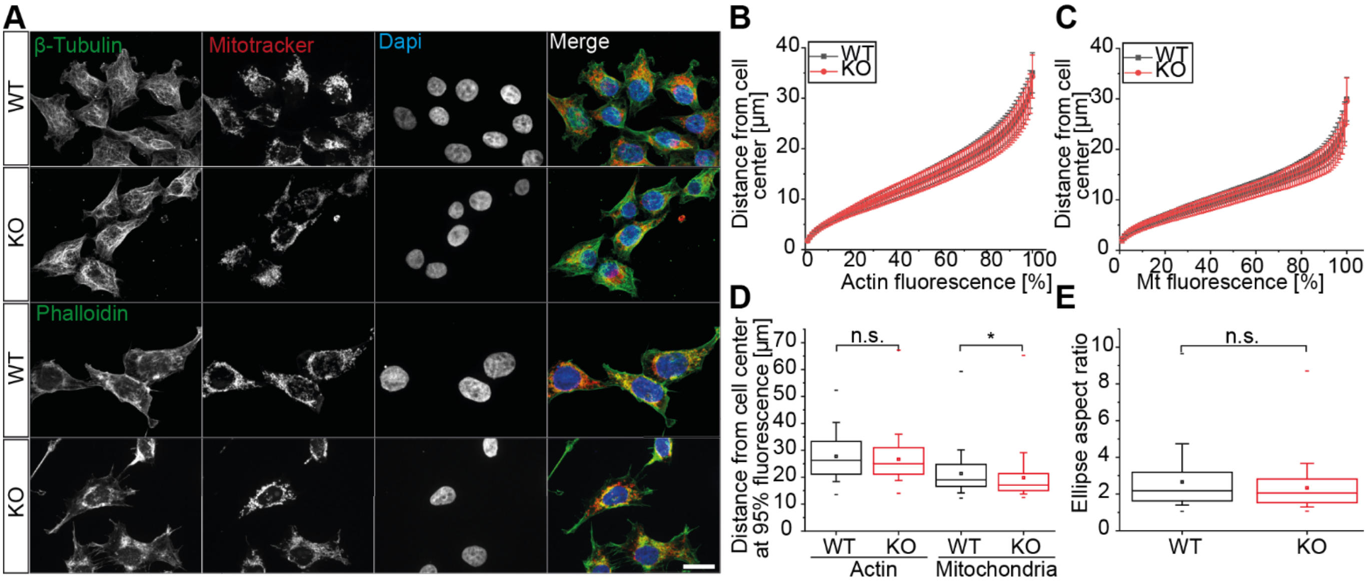

Fig. S1 Deletion of Myo19 does not influence the microtubule or actin organization in the cell. (A) Representative images of WT and Myo19-KO cells stained with Mitotracker, β-tubulin antibody or FITC-Phalloidin and DAPI. Quantification of the fluorescence as a function of the distance from the cell center for F-actin (B) and mitochondria (C) in Myo19-KO and WT cells. (D) Statistical analysis of the distance from the center of the cell at 95% of total fluorescence for F-actin and mitochondria. The median of at least 90 cells is plotted with the corresponding 25th and 75th percentile. (E) Ellipse aspect ratio values as a proxy for cell shape showed no significant difference between WT and Myo19-deficient cells. * p ≤0.05, n.s., not significant. Scale bar, 20 µm.

Journal of Cell Science • Supplementary information

Fig. S2

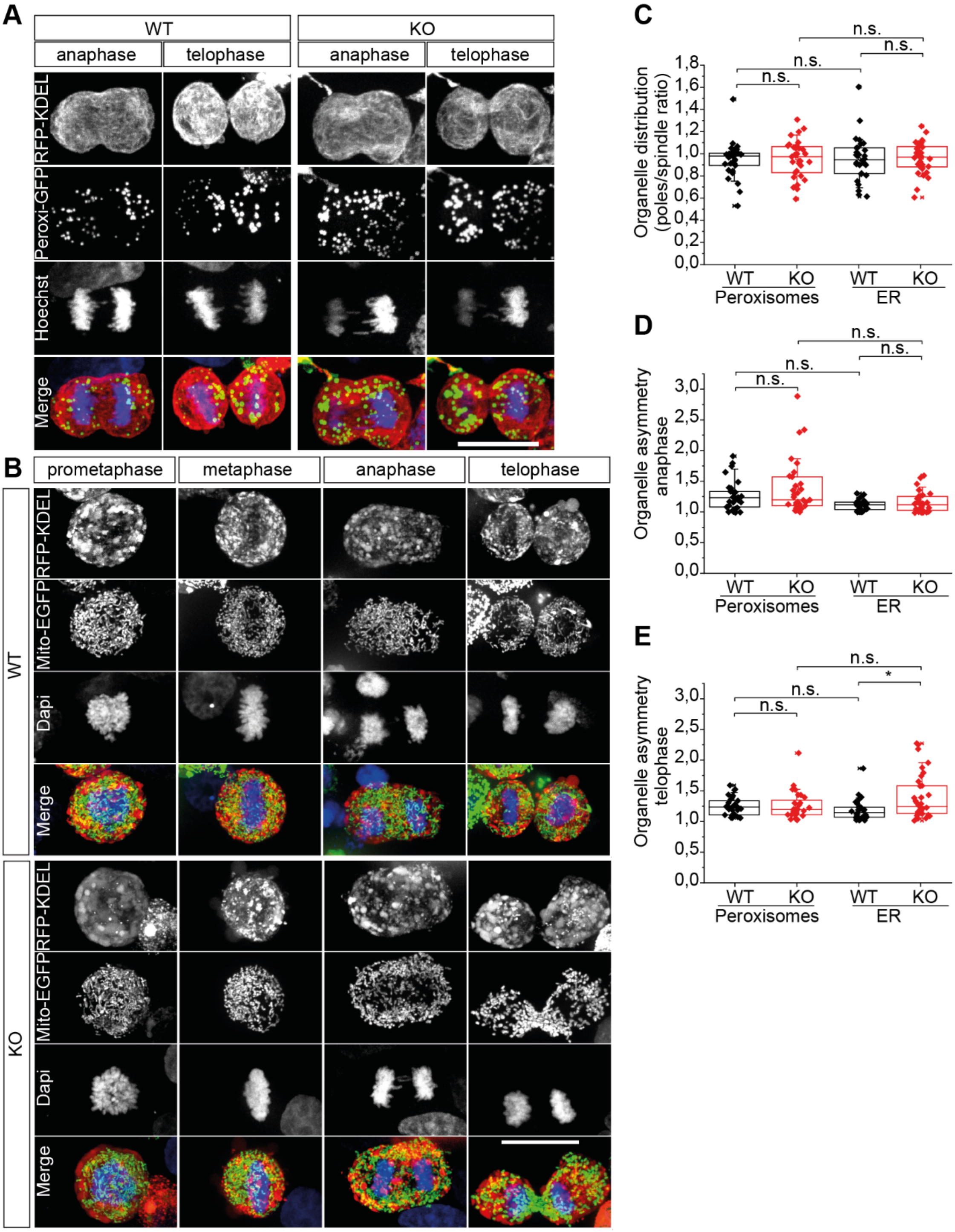

Fig. S2 Distribution of peroxisomes and ER during mitosis in Myo19-KO cells. (A) Representative images of cells at anaphase and telophase showing ER (RFP-KDEL, red), peroxisomes (Peroxi-GFP, green) and chromosomes (Hoechst). Scale bar, 20 µm. (B) ER and mitochondria organization in HEK WT and Myo19-KO cells was assessed by cotransfection with RFP-KDEL and Mito-EGFP. Different mitotic stages were determined by staining with DAPI. Scale bar, 20 µm. (C) Quantification of

organelle distribution at cell poles/spindle at anaphase. (D, E) Quantification of the asymmetry in organelle distribution in cells at anaphase and telophase. Data are displayed as box plots with the corresponding 75th and 25th percentile and the median. The minimum and maximum values are indicated as dashes. Data are from at least 26 cells per cell clone. * $p \leq 0.05$; n.s., not significant. Wild-type (WT) and Myo19-KO cells (KO).

Fig. S3

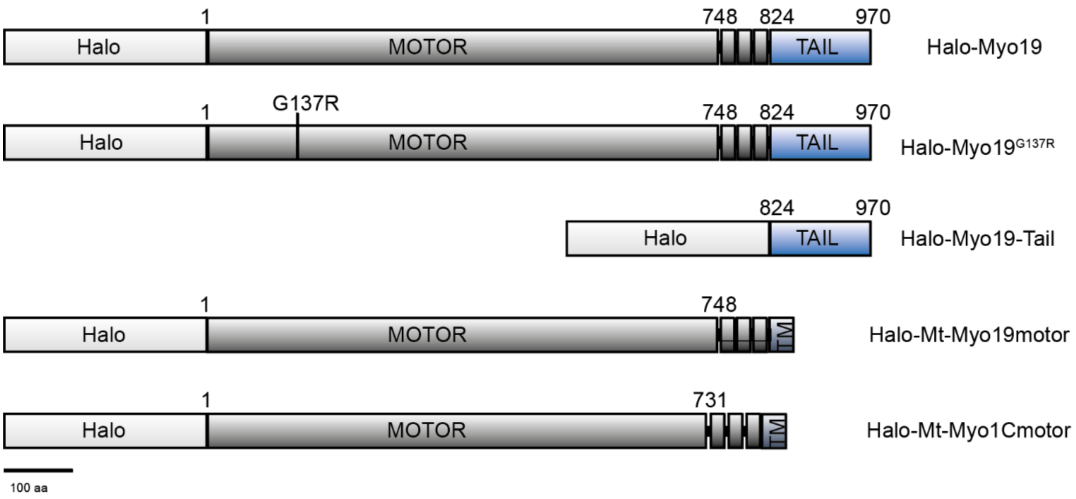

Fig. S3: Schematic representation of Halo-tagged Myo19 and Myo1c protein constructs used in this study. TM: transmembrane domain of Miro1; Halo-tag was fused the N-terminus of all constructs. Numbers indicate amino acid residues.

Journal of Cell Science • Supplementary information

Fig. S4

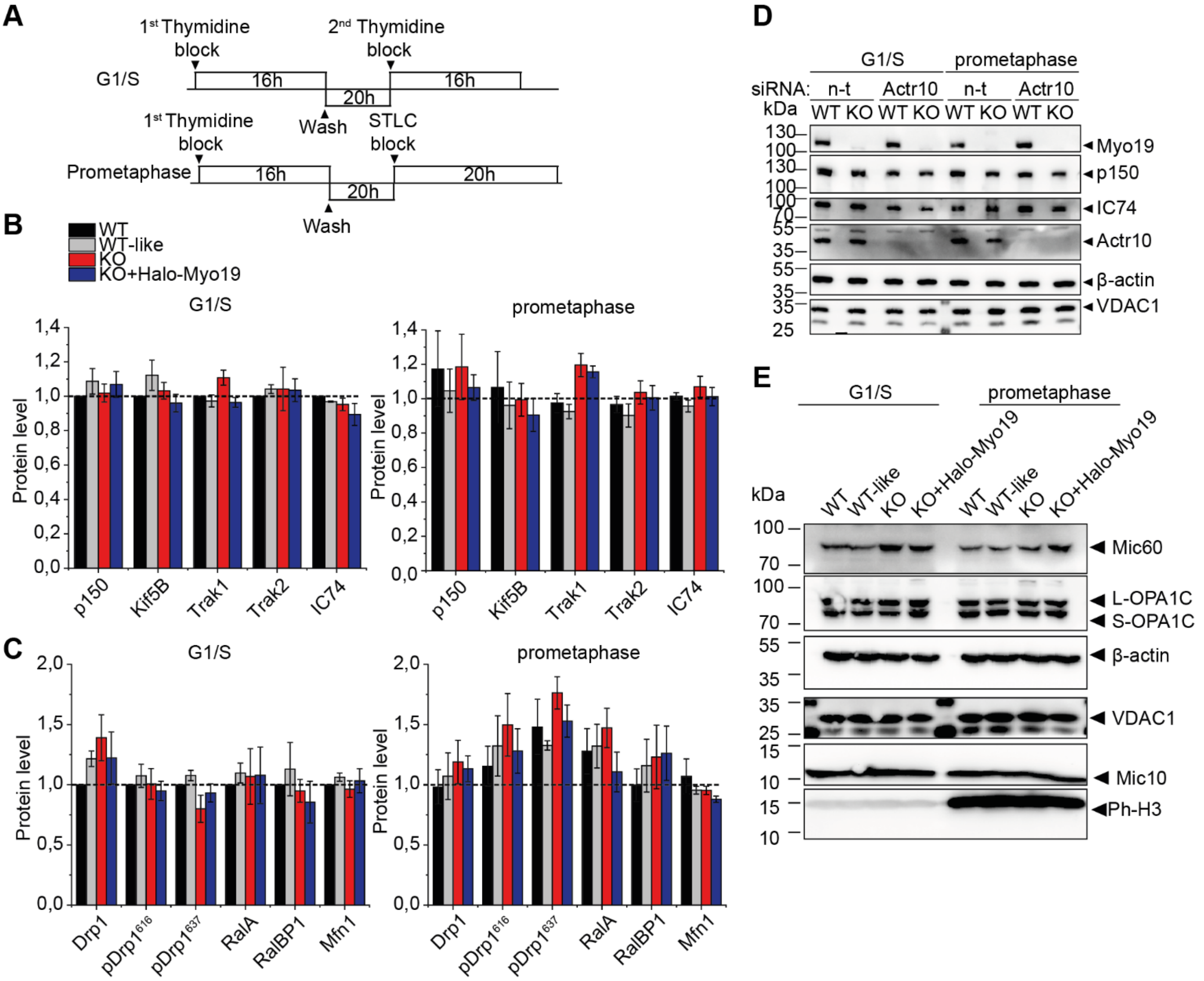

Fig. S4: Myo19-knockout cell lines showed no differences in the total levels of microtubule-based molecular motors nor fission and fusion related proteins at both interphase and prometaphase. (A) Scheme of cell synchronization at interphase and prometaphase of the cell cycle. (B,C) Quantification of protein levels in the cytosol of wild-type (WT), wild-type-like (WT-like), Myo19 knockout (KO) and Myo19-KO expressing Halo-Myo19 (KO+Halo-Myo19) cells normalized to GAPDH or VDAC1 as fold change compared to WT at G1/S and prometaphase. Data are from 3-7 independent experiments; error bars represent ±SEM. (D) Immunoblot of protein levels of cells treated with non-targeting (n-t) or Actr10 siRNA and synchronized at interphase or prometaphase. (E) Immunoblot of protein levels in cell homogenates of cells synchronized at interphase or prometaphase.

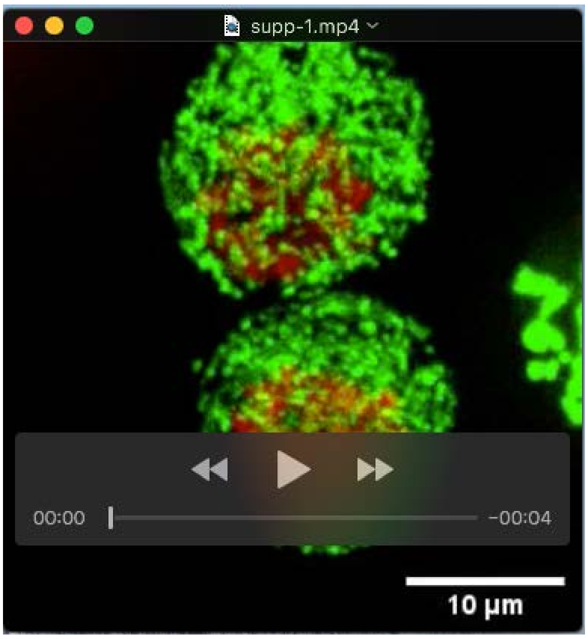

Movie 1

Example of subcellular mitochondria distribution during mitosis and cytokinesis in HEK WT cells. Fluorescence images were taken every 6 minutes of cells released from prometaphase (STLC block) showing mitochondria (Mito-EGFP, green) and chromosomes (H2B-mCherry, red). Scale bar, 10 µm.

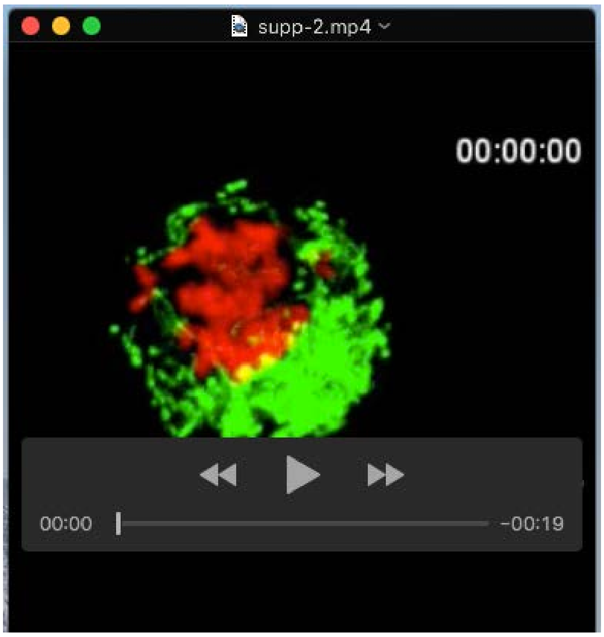

Movie 2

Example of subcellular mitochondria distribution during mitosis and cytokinesis in HEK Myo19-KO cells that divided successfully. Fluorescence images were taken every 6 minutes of cells released from prometaphase (STLC block) showing mitochondria (Mito-EGFP, green) and chromosomes (H2B-mCherry, red). Scale bar, 10 µm.

Journal of Cell Science • Supplementary information

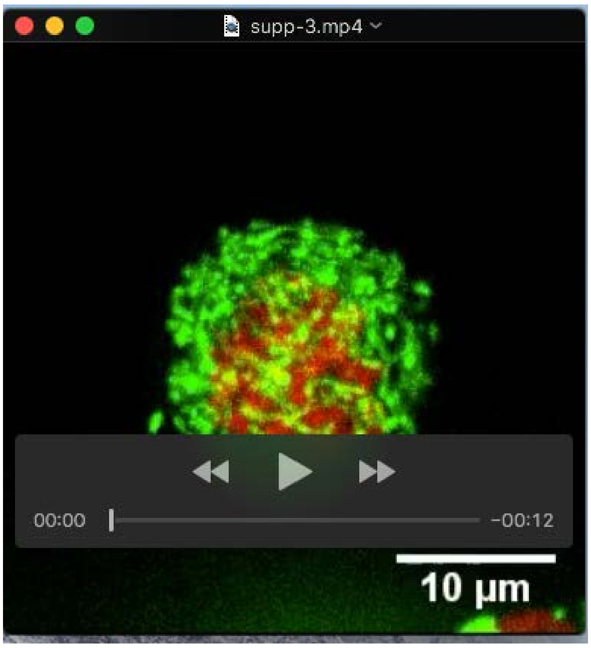

Movie 3

Example of subcellular mitochondria distribution during cell division in Myo19-KO cell that failed to divide (from Figure 1 E). Fluorescence images were taken every 6 minutes of cells released from prometaphase (STLC block) showing mitochondria (Mito-EGFP, green) and chromosomes (H2B-mCherry, red). Scale bar, 10 µm.

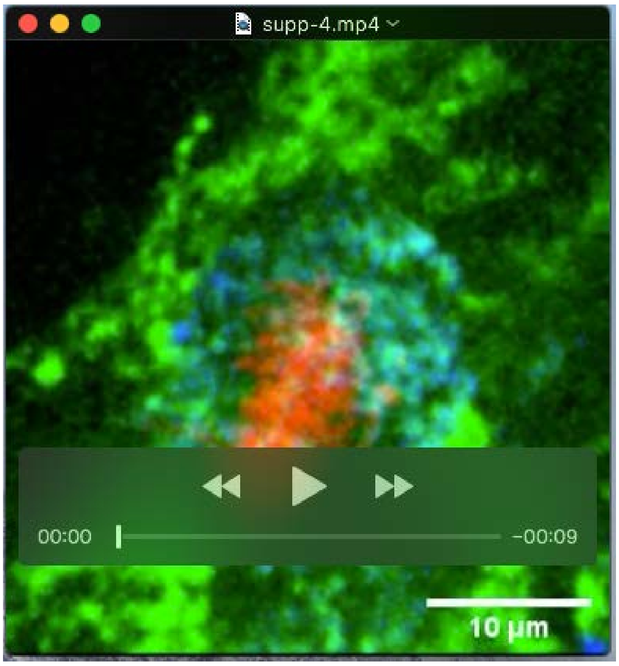

Movie 4

Myo19 remains associated with mitochondria during mitosis. Example of cells stably expressing Halo-Myo19 that undergo mitosis and cytokinesis. Cells were transfected with Mito-BFP, H2B-mCherry and stained with R110Direct-ligand. Images were taken every 6 minutes. Scale bar, 10 µm.

Journal of Cell Science • Supplementary information

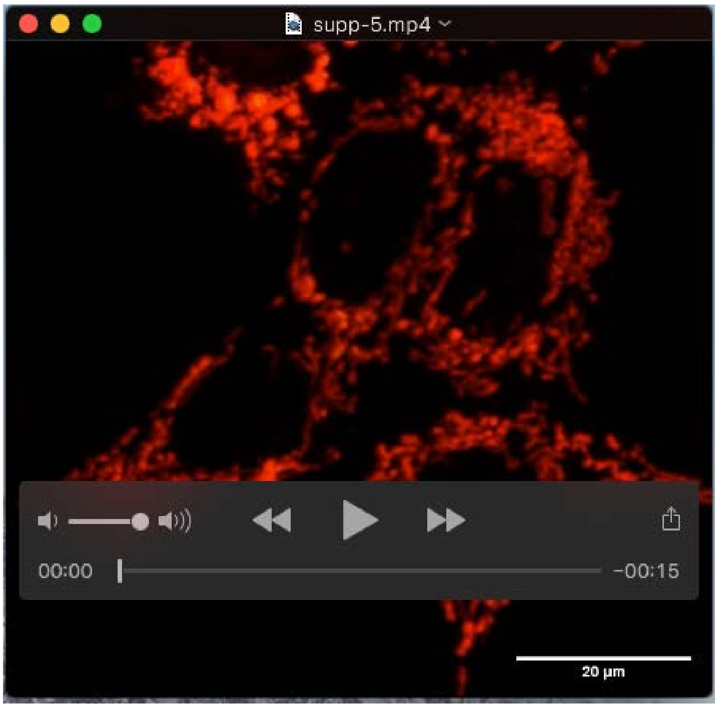

Movie 5

Live-cell imaging of WT cells at interphase expressing photoactivatable Mito-GFP (PA-GFP-Mito, green). Mitochondria were stained with Mitotracker Orange (red). Video frames were taken before and immediately after photoactivation of a ROI and then every 30 s for 20 min. Scale bar, 10 µm.

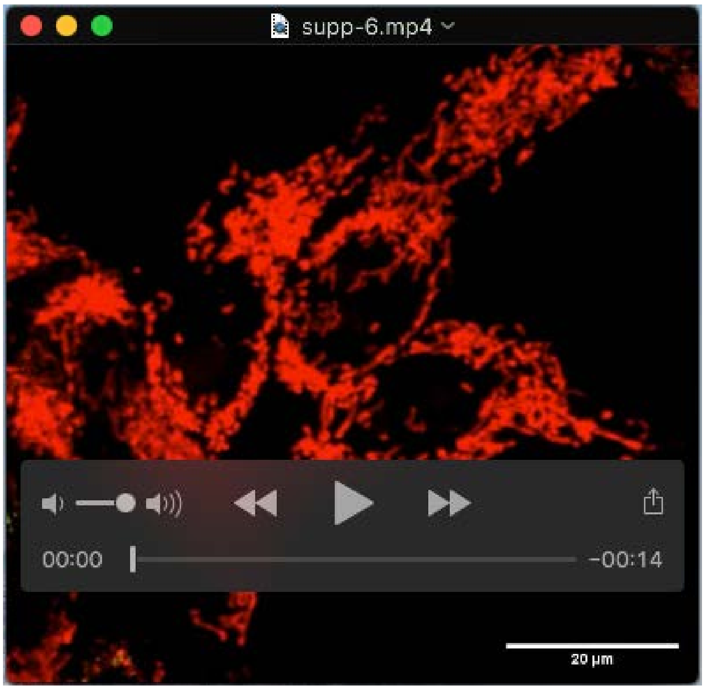

Movie 6

Live-cell imaging of Myo19-KO cells (KO) at interphase expressing photoactivatable Mito-GFP (PA-GFP-Mito, green).. Mitochondria were stained with Mitotracker Orange (red). Video frames were taken before and immediately after photoactivation of a ROI and then every 30 s for 20 min. Scale bar, 10 µm.

Journal of Cell Science • Supplementary information

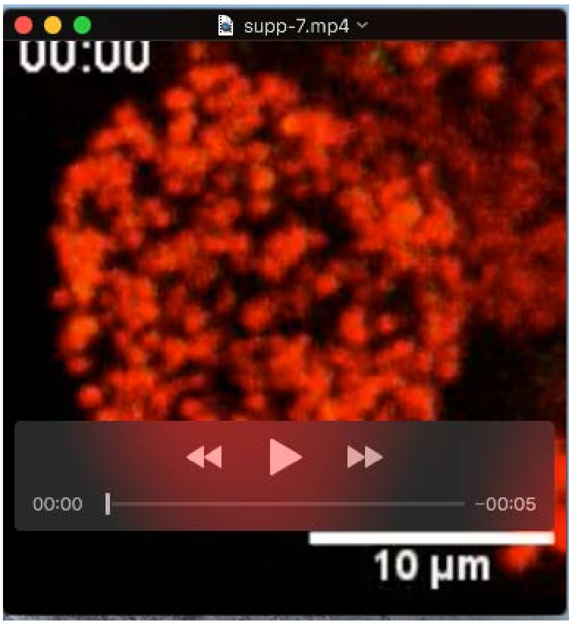

Movie 7

Live-cell imaging of WT cells at prometaphase expressing photoactivatable Mito-GFP (PA-GFP-Mito, green).. Mitochondria were stained with Mitotracker Orange (red). Video frames were taken before and immediately after photoactivation of a ROI and then every 30 s for 20 min. Scale bar, 10 µm.

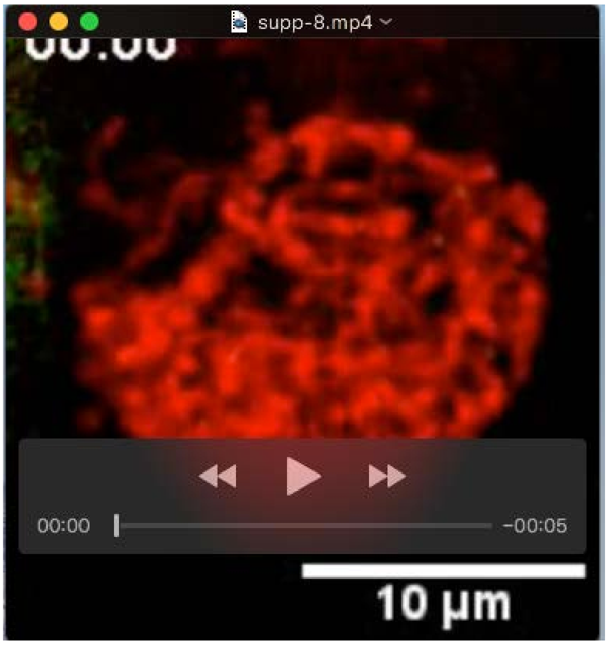

Movie 8

Live-cell imaging of Myo19-KO cells (KO) prometaphase expressing photoactivatable Mito-GFP (PA-GFP-Mito, green). Mitochondria were stained with Mitotracker Orange (red). Video frames were taken before and immediately after photoactivation of a ROI and then every 30 s for 20 min. Scale bar, 10 µm.

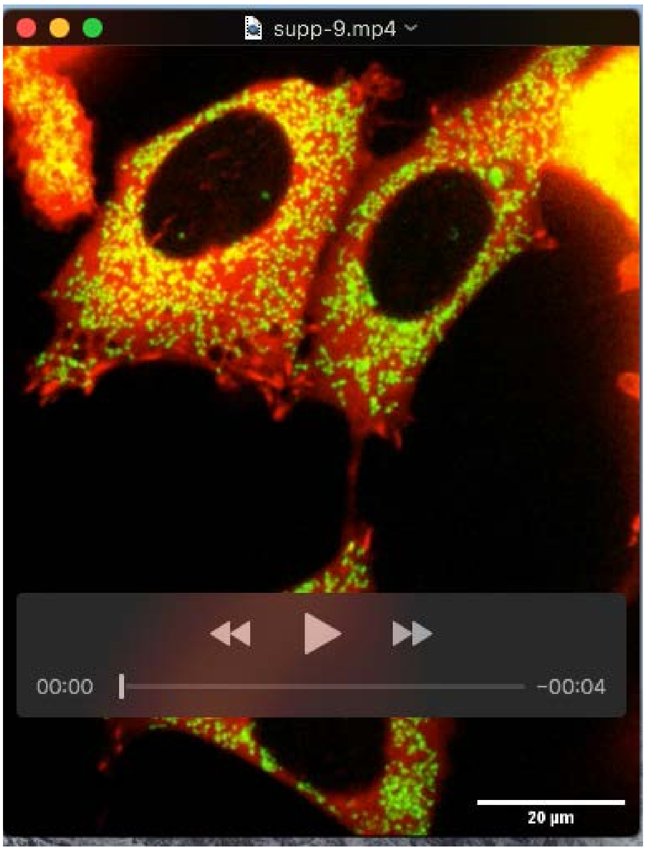

Movie 9

Examples of interplay between mitochondria and focal adhesions in WT cells that were transiently transfected with mCherry-Paxillin and Mito-EGFP. Images were taken for 30 min with a frame rate of 1 min. Scale bar, 20 μm.

Journal of Cell Science • Supplementary information

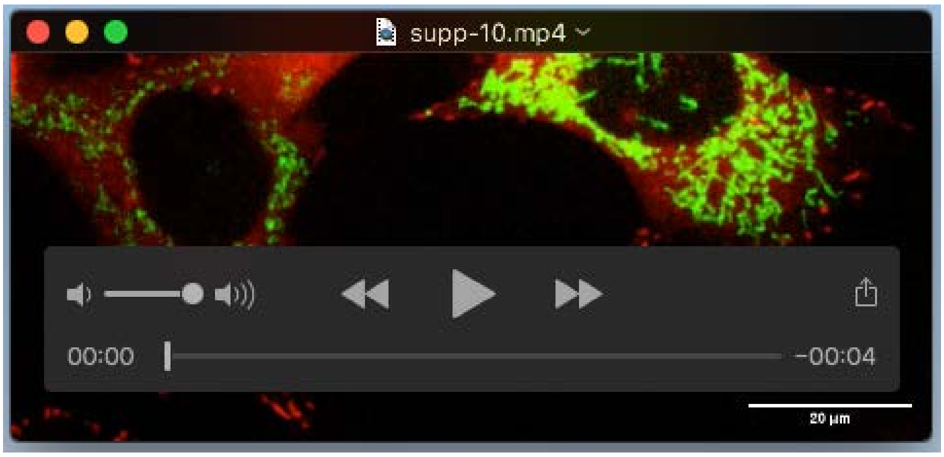

Movie 10

Examples of interplay between mitochondria and focal adhesions in WT cells that were transiently transfected with mCherry-Paxillin and Mito-EGFP. Images were taken for 30 min with a frame rate of 1 min. Scale bar, 20 µm.

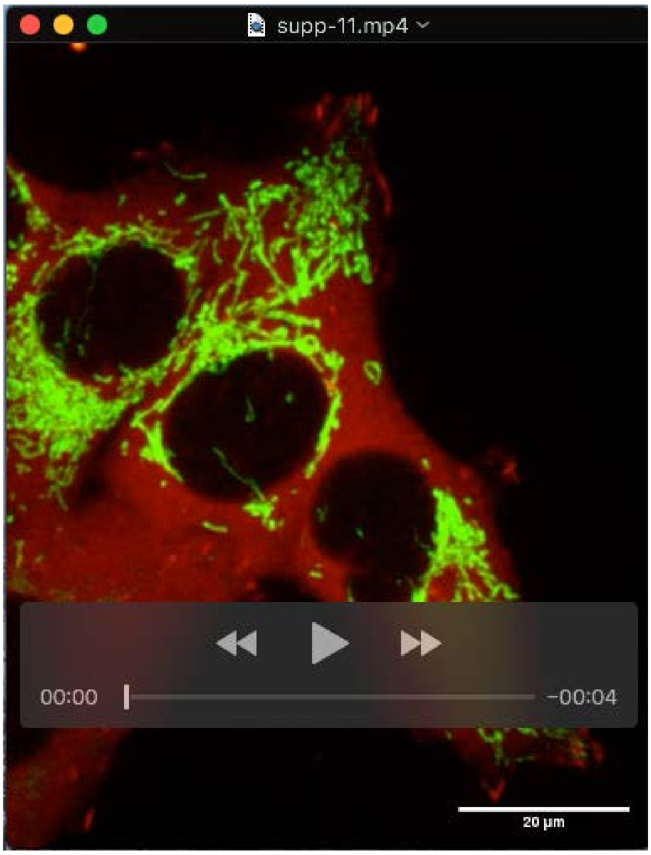

Movie 11

Examples of interplay between mitochondria and focal adhesions in Myo19-KO cells that were transiently transfected with mCherry-Paxillin and Mito-EGFP. Images were taken for 30 min with a frame rate of 1 min. Scale bar, 20 µm.

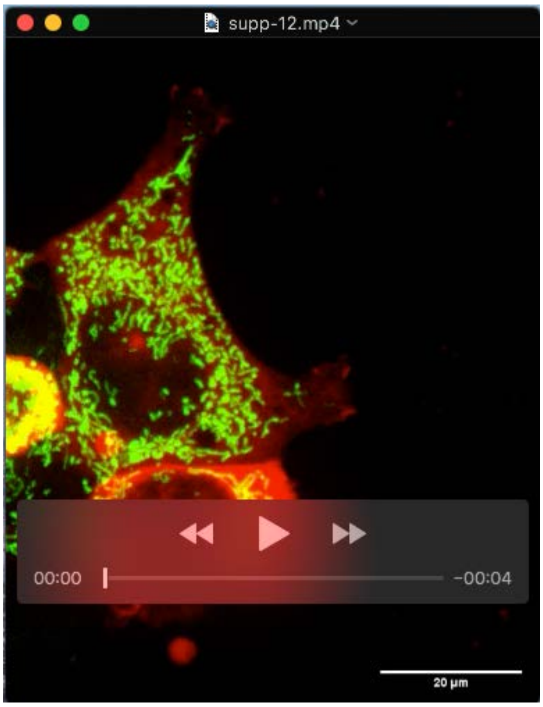

Movie 12

Examples of interplay between mitochondria and focal adhesions in Myo19-KO cells that were transiently transfected with mCherry-Paxillin and Mito-EGFP. Images were taken for 30 min with a frame rate of 1 min. Scale bar, 20 µm

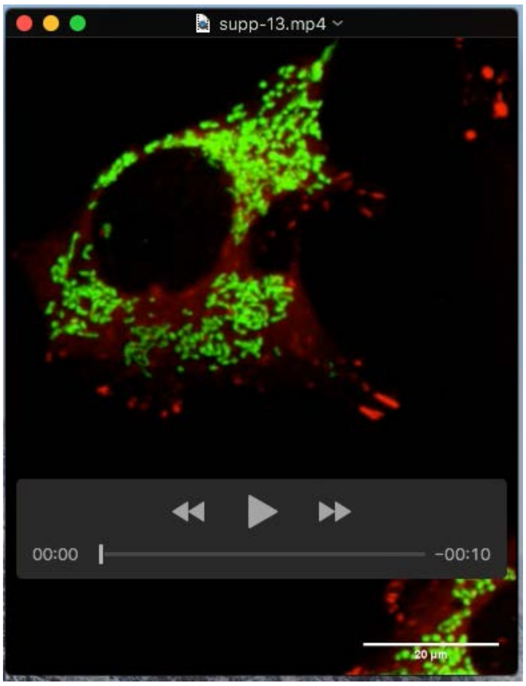

Movie 13

Examples of interplay between mitochondria and focal adhesions in Myo19-KO cells expressing Halo-Myo19 that were transiently transfected with mCherry-Paxillin and Mito-EGFP. Images were taken for 30 min with a frame rate of 1 min. Scale bar, 20 µm.

Journal of Cell Science • Supplementary information
