# Supplementary material for: Coordination of mitochondrial and cellular dynamics by the actin-based motor Myo19
Source: J Cell Sci. 2021 May 20;134(10):jcs255844. doi: 10.1242/jcs.255844 (PMC8186483; doi:10.1242/jcs.255844)
Supplement: Reviewer comments [file joces-134-255844_review_history.pdf]

Journal of Cell Science

# Coordination of mitochondrial and cellular dynamics by the actin-based motor Myo19

Katarzyna Majstrowicz, Ulrike Honnert, Petra Nikolaus, Vera Schwarz, Stefanie J. Oeding, Sandra A. Hemkemeyer and Martin Bäehler
DOI: 10.1242/jcs.255844

**Editor**: Michael Way

**Review timeline**

| | |
|---|---|
| Original submission: | 15 October 2020 |
| Editorial decision: | 12 November 2020 |
| First revision received: | 9 March 2021 |
| Editorial decision: | 1 April 2021 |
| Second revision received: | 9 April 2021 |
| Accepted: | 12 April 2021 |


---

**Original submission**

First decision letter

MS ID#: JOCES/2020/255844

MS TITLE: Coordination of mitochondrial dynamics by the actin-based motor Myo19 and microtubule-based motors during interphase and mitosis

AUTHORS: Katarzyna Majstrowicz, Ulrike Honnert, Petra Nikolaus, Vera Schwarz, Stefanie Jennifer J Oeding, Sandra A Hemkemeyer, and Martin Baehler
ARTICLE TYPE: Research Article

We have now reached a decision on the above manuscript.

To see the reviewers' reports and a copy of this decision letter, please go to: https://submit-jcs.biologists.org and click on the 'Manuscripts with Decisions' queue in the Author Area. (Corresponding author only has access to reviews.)

As you will see, the reviewers raise a large number of criticisms and comments that prevent me from accepting the paper at this stage. They suggest, however, that a revised version might prove acceptable, if you can address their concerns. I think the non-mitochondrial role for Myo19 raised by reviewer 1 should be explored or at least acknowledged. As suggested by reviewer 2 you may also want to remove the peroxisome data as the link with Myo19 is not immediately obvious. If you think that you can deal satisfactorily with the criticisms on revision, I would be pleased to see a revised manuscript. We would then return it to the reviewers.

*We are aware that you may be experiencing disruption to the normal running of your lab that makes experimental revisions challenging. If it would be helpful, we encourage you to contact us to discuss your revision in greater detail. Please send us a point-by-point response indicating where you are able to address concerns raised (either experimentally or by changes to the text) and where you will not be able to do so within the normal timeframe of a revision. We will then provide further guidance. Please also note that we are happy to extend revision timeframes as necessary.*

© 2021. Published by The Company of Biologists under the terms of the Creative Commons Attribution License (https://creativecommons.org/licenses/by/4.0/).

Please ensure that you clearly highlight all changes made in the revised manuscript. Please avoid using 'Tracked changes' in Word files as these are lost in PDF conversion.

I should be grateful if you would also provide a point-by-point response detailing how you have dealt with the points raised by the reviewers in the 'Response to Reviewers' box. Please attend to all of the reviewers' comments. If you do not agree with any of their criticisms or suggestions please explain clearly why this is so.

Reviewer 1

*Advance summary and potential significance to field*

The manuscript by Majstrowicz et al. characterises several aspects of a Myo19 knockout cell line. The authors confirm a previous result that loss of Myo19 leads to asymmetric mitochondrial segregation during mitosis and subsequent multinucleation. Alongside these confirmatory results, the authors characterise a variety of cellular parameters including mitochondrial oxygen consumption, focal adhesions and peroxisomal motility, finding potential dysfunction in these processes. The work is well presented and, for the most part, properly controlled.

*Comments for the author*

An important claim of the paper is that the absence of Myo19 on mitochondria causes the observed phenotypes. However, while most Myo19 is visibly on mitochondria, it is possible that the small non-mitochondrial pool of Myo19 is responsible for the phenomena herein. It is therefore important to ascertain the mitochondrial specificity of the phenotypes and/or tone down the claims.

1. Whilst there is convincing asymmetry in mitochondrial segregation in the Myo19 KO cells, it is not clear that this is specific to the mitochondria. It is possible that most cytoplasmic contents are asymmetrically segregated. This could be the case if cell division became intrinsically asymmetric in the absence of Myo19. Indeed, the representative images in Figure 4I appears to show that the ER is asymmetrically segregated. Controls to show the mitochondrial specificity of this phenotype are required.
2. The data contained in Figure 2, whereby artificially forcing Myo19 on mitochondria does not rescue any of the phenotypes characterised in the Myo19 KO cells supports the idea that the phenotypes are due to the absence of a small pool of non-mitochondrial Myo19. If, as questioned in Point 1, many cytoplasmic contents are asymmetrically segregated, this would add further weight to this idea. It may be, understandably, difficult to counter this idea experimentally. A better acknowledgment of this possibility (e.g. in the discussion) might be required.
3. The seahorse data show that the all respiratory rates are lower in Myo19 KO cells. However these data take into account a fixed number of cells, not a fixed amount of mitochondria. While this might indicate less mitochondrial mass per cell, it could also mean cells are smaller. In fact, Figure 5F appears to support the idea that mitochondrial content in the cell is unchanged, as VDAC1 and the complex components do not change when normalised to actin or GAPDH. The mitochondria-centric interpretation again does not consider the possibility that yo19 might act on other cellular structures.
4. The lower number of focal adhesions (FA) is interpreted in a mitochondria-centric way as the result of decreased ATP production. If true, this decrease in ATP should be measurable, any condition causing the same reduction in ATP should yield the same FA phenotypes, and most importantly these phenotypes should be rescuable by tinkering with the energy status of the cell to increase ATP (or with extracellular ATP https://jcs.biologists.org/content/132/7/jcs223925, or phosphocreatine?). Another possibility is that a non-mitochondrial pool of Myo19 accounts for the phenotypes observed. After all, actin dynamics are required for focal adhesion and heavily influenced by myosins. So this whole part is too preliminary to be conclusive.
5. The authors state “that the loss of Myo19 is not affecting ER morphology and the regulation of mitochondrial fusion/fission by Myo19 is not ER-dependent”. There is insufficient evidence to support this claim. Firstly, the staining presented would make it very difficult to ascertain if there were any morphological changes in the ER. More importantly, however, the role of the ER in mitochondrial fission is elicited through ER-mitochondria contact sites, which the authors do not study.

© 2021. Published by The Company of Biologists under the terms of the Creative Commons Attribution License (https://creativecommons.org/licenses/by/4.0/).

Minor comments
1. Some of the image labels in Figure 4I are not readable.
2. What is “directionality” defined as with regards to peroxisomal motility?

Reviewer 2

*Advance summary and potential significance to field*

The manuscript by Majstowicz et al. describes the characterisation of a MYO19 KO in HEK293T cells using CRISPR/cas9. The MYO19 KO cells display a range of often seemingly unrelated phenotypes ranging from defects in cell division and changes in mitochondria distribution and morphology in mitotic cells to defects in mitochondrial activity in interphase cells, a reduction in focal adhesions and finally peroxisome velocity. While some of these phenotypes can be linked directly to the absence of MYO19, others appear to be more of an undefined indirect nature.
For a coherent narrative, the paper should focus on a detailed analysis of one of these observed phenotypes. The main body of work is on the role of MYO19 regulating mitochondria distribution in mitotic cells, however, a manuscript describing these findings has already been published by Rohn et al. Current Biology 2014.

*Comments for the author*

My main concerns are:
1. Overall, this manuscript contains a large body of work and detailed quantifications of phenotypes, however, it is a list of findings and fails to connect the separate observations into one story furthering our understanding on the cellular functions of MYO19. I would recommend to remove the last figure and extend the findings on the impact of MYO19 depletion on peroxisome motility in a separate manuscript.
2. Unfortunately, some of the main findings, in particular the cell division defect and the role of MYO19 for mitochondria distribution during mitosis, has already been shown by Rohn et al. 2014.
3. Although it is very recommendable that the authors have made great efforts to quantify their observations, the statistical analysis is either completely missing for crucial experiments or lacking detail (is it the mean ±SEM that has been plotted or mean ± SD?). In addition, for multiple comparisons as is often the case in this manuscript, a Student’s t-test or Mann-Whitney test are not appropriate and instead an ANOVA with post-hoc test, such as Bonferroni, should be used. Most importantly, ‘n’ does not represent the number of cells analysed in each experiment, but is the number of independent repeats of each experiment. This is particularly confusing as, for example, in Figure 4B-E four points are plotted but the n number is stated as 90.

In figure 1A the MYO19 KO cells were analysed by immunoblotting, which shows still a band at the size of MYO19 in the KO cells. Is this still a mixed clone of KO and WT cells?
The results in figure 1 C, C’, D and D’ describe that absence of MYO19 leads to division failure causing an increase in multinucleated cells. The importance of MYO19 for cell division has already been shown in Rohn et al., 2014. The same paper also reported previously that in MYO19 siRNA KD cells mitochondria are asymmetrically distributed and accumulate inappropriately at spindle poles during anaphase, which are very similar to the results shown in figure 1 E, F, G and H.

In figure 2 the authors perform rescue experiments using wildtype and mutant forms of MYO19 and show that the phenotypes described in figure 1 and by Rohn et al. can only be rescued by fully functional MYO19. This is a valuable control experiment, but does not give any new insights into the cellular functions for MYO19.

The results in figure 3 show that MYO19 remains associated with mitochondria during mitosis (figure 3 A-C) and that in MYO19 KO cells slightly increased levels of dynactin and TRAK1 are present on mitochondria. The authors than use two different approaches to inhibit dynein activity by either using the drug Dynarrestin or by depleting a subunit of the dynactin complex. Depletion of the dynactin subunit is able to prevent polewards movement of mitochondria, suggesting that dynein activity is inhibited and required for mitochondrial transport towards the minus end of microtubules at the spindle poles. Dynarrestin, however, leads to an increase in localisation of mitochondria at spindle poles in wildtype cells? This requires an explanation.

© 2021. Published by The Company of Biologists under the terms of the Creative Commons Attribution License (https://creativecommons.org/licenses/by/4.0/). 

In figure 4 the authors present results on the impact on MYO19 KO on mitochondria morphology. The authors describe that in MYO19 KO cells “the number of mitochondrial individuals and networks was decreased” and “additionally a highly interconnected network with more branching was revealed”. This seems to be very contradictory. Please explain.

In an attempt to explain a link between MYO19 KO and changes in mitochondria morphology, the authors perform cellular fractionation experiments and identified a very slight increase in DRP1 levels associated with mitochondria in MYO19 KO cells; the change in DRP1 levels as shown on the blot is not convincing. Thus, it is crucial to include the correct statistical analysis and p-value in figure 4 L.

In the next figure the authors switch to investigate mitochondrial function not in mitotic cells but during interphase, which is not linked to the experiments performed in figure 1-4. The authors identify changes in mitochondrial mass between WT and KO cells by flow cytometry using two different dyes, however, the differences were dismissed as no changes in protein levels of the various OXPHOS complexes were observed. Why can the flow cytometry measurements be ignored?

In the next set of experiments the authors change focus again and demonstrate the loss of MYO19 causes a reduction in cell adhesion. No obvious link between MYO19 – mitochondria and focal adhesions was observed, only a small change in expression levels of focal adhesion associated proteins such as vinculin and paxillin.

In the final figure the authors describe a change in peroxisome velocity in MYO19 KO cells, which is an interesting observation, since MYO19 cannot be localised to peroxisomes. However, this set of experiments again is a stand-alone observation, which is not linked to either the role of MYO19 during mitochondria distribution during mitosis, for regulating mitochondria activity or for a potential link of MYO19 to focal adhesions.

Further points:
The title is not capturing the focus of the manuscript, since microtubule dynamics is not analysed in interphase cells and no insight in the role of microtubule motors is provided.
An overview on the observed phenotype and proposed roles of MYO19 would be very helpful.

Reviewer 3

*Advance summary and potential significance to field*

See below

*Comments for the author*

Myo19 is an actin-based motor protein that localizes to mitochondria and there is much interest in its functions. Recent results from the Baehler group and others demonstrate that mitochondrial GTPase Miro acts as Myo19 receptor and are especially interesting given previous work reporting that Miro recruits the microtubule-based motor proteins kinesin and dynein to mitochondria. In the current manuscript, Majstrowicz et al use HEK cells and CRISPR to generate the first Myo19 KO cells and investigate their phenotypes. The authors begin by showing that Myo19 KO results in a modest asymmetry in the distribution of mitochondria in anaphase and telophase, confirming the knockdown results in HeLa cells reported by Rohn et al. Majstrowicz et al also confirm that Myo19 KO increases the number of multinucleated cells, but they extend this result by generating a panel of stable cell lines to show that full length Myo19 rescues these defects while a motor domain mutation, the Myo19 tail alone, or the Myo19 motor alone do not. A key result is the data showing that Myo19 KO decreases ATP production by 40%. This exciting result is novel and unexpected and demonstrates that mitochondria require Myo19 to maintain normal levels of ATP production. The authors also found that Myo19 KO cells detach from the substrate more frequently than controls, have lower levels of phosphorylated paxillin, have a decreased number of focal adhesions per cell, and have a decreased average size of focal adhesions. The authors conclude by reporting that

© 2021. Published by The Company of Biologists under the terms of the Creative Commons Attribution License (https://creativecommons.org/licenses/by/4.0/). 

peroxisomes, a class of mitochondria related organelle, do not colocalize with Myo19, but do move ~13% slower in KO cells.
This manuscript's generation of Myo19 KO cells and their cell biological defects will be of interest both to those studying motor proteins and to those investigating mitochondrial physiology. The data showing that Myo19 KO decreases ATP production by 40% is particularly important and interesting.

Specific comments:
1: Because Myo19 KO resulted in a 40% decrease in ATP production, it seems possible that some of the changes the authors report are due to the decreased ATP levels or changed ATP/ADP ratios that would secondarily affect the activity of other motor proteins and/or kinases. The authors should mention this possibility in their discussion, especially given the relatively modest effects of Myo19 KO on peroxisome motility.

2: The paper by Bocanegra et al (2019) reporting Miro-dependent and Miro-independent mechanisms of Myo19 interaction with mitochondrial membranes should be cited in the introduction or discussion. Given that KO of the actin-based motor Myo19 perturbs the distribution of mitochondria during mitosis the authors should also consider citing the recent MBOC paper by AM Kita et al that demonstrates the presence of F-actin in vertebrate spindles.

3: To unambiguously specify the constructs used here whose sequences have not already been published or deposited in Addgene, the authors should include the full nucleotide sequences of the constructs they generated in the supplemental material. This would also enhance reproducibility by providing a permanent record of the exact vector and insert sequences, along with any linker sequences, splice forms, or sequence variants.

Minor comments:
70: Add MEF to the list of abbreviations.

106: CRIPR should be CRISPR

109: Did the phenotype of the CRISPR Myo19-KO cells weaken with the number of passages? If so, this should be mentioned, and the range of passages used for the experiments should be stated.

120: The authors should clarify what they mean by "struggled to divide".

126: For each mean reported here and in the rest of the manuscript, the authors should include the standard deviation or SEM.

140: Is the G137R mutation expected to result in a mutant that cannot bind to any nucleotide and is trapped in rigor? This should be mentioned and could result in an unnatural gain-of-function phenotype where mitochondria are anchored to actin.

199: This sentence is not clear.

267-268: The last part of this sentence needs to be changed. The authors show that overall ER morphology is independent of Myo19, but they do not show that Myo19 regulation of mitochondrial fusion/fission is independent of ER--this would require perturbing or eliminating the ER.

281: It would be more accurate to state that no differences in phosphorylation were detected.

306: Add FCCP to the list of abbreviations and provide more background on the experiments in Figure 5H-I and the use of oligomycin, FCCP, and rotenone.

356: The authors should specify if the mean velocity reported here corresponds to the net displacement divided by the 2 minute recording time, the mean of ~100 nm displacements recorded once per second, or something else. Because velocity is a vector quantity and speed is scalar, it is important to know how movements in opposite directions were dealt with when calculating the mean.

538: Zhang et al., 2014 is missing from the references.

© 2021. Published by The Company of Biologists under the terms of the Creative Commons Attribution License (https://creativecommons.org/licenses/by/4.0/).

661: State whether the image analyses and measurements were performed blinded.

---

**First revision**

Author response to reviewers' comments

Reviewer 1:

An important claim of the paper is that the absence of Myo19 on mitochondria causes the observed phenotypes. However, while most Myo19 is visibly on mitochondria, it is possible that the small non-mitochondrial pool of Myo19 is responsible for the phenomena herein. It is therefore important to ascertain the mitochondrial specificity of the phenotypes and/or tone down the claims.

There is currently no evidence for a small non-mitochondrial pool of Myo19 under physiological conditions. When Myo19 is released from mitochondria (from Miro) it is not stable and gets rapidly degraded (Lopez-Domenech et al., 2018; Oeding et al., 2018). Therefore, it seems unlikely that a cytoplasmic pool of Myo19 is responsible for the observed phenotypes. The observed phenomena can be traced back to mitochondrial alterations.

1. Whilst there is convincing asymmetry in mitochondrial segregation in the Myo19 KO cells, it is not clear that this is specific to the mitochondria. It is possible that most cytoplasmic contents are asymmetrically segregated. This could be the case if cell division became intrinsically asymmetric in the absence of Myo19. Indeed, the representative images in Figure 4I appears to show that the ER is asymmetrically segregated. Controls to show the mitochondrial specificity of this phenotype are required.

The distribution of peroxisomes and ER was investigated in HEK WT and Myo19-KO cells. The analysis showed that the distribution of these two organelles was not significantly altered at anaphase when Myo19 was missing. However, the distribution of the ER, but not of the peroxisomes, was significantly asymmetrical at telophase in Myo19-KO cells. The data were newly included in Fig. S2.

2. The data contained in Figure 2, whereby artificially forcing Myo19 on mitochondria does not rescue any of the phenotypes characterised in the Myo19 KO cells supports the idea that the phenotypes are due to the absence of a small pool of non-mitochondrial Myo19. If, as questioned in Point 1, many cytoplasmic contents are asymmetrically segregated, this would add further weight to this idea. It may be, understandably, difficult to counter this idea experimentally. A better acknowledgment of this possibility (e.g. in the discussion) might be required.

The observation that forcing the Myo19 motor domain onto mitochondria does not rescue the mitochondrial phenotype during mitosis and cytokinesis is indeed a very interesting point, because the phenotype has been explained by a loss of an association of mitochondria with the actin cytoskeleton. However, this assumption is not supported by our finding, provided the mitochondria-linked motor region is working as when it is free in solution. How a small cytosolic pool of Myo19 could affect mitochondria distribution during mitosis would need some novel creative ideas. An obvious explanation for the mitotic phenotype in Myo19-deficient cells could be that the activities of microtubule-based motors and Myo19 need to be coordinated. We acknowledge these possibilities in the Discussion. A cytosolic pool of Myo19 does not need to be implied.

3. The seahorse data show that the all respiratory rates are lower in Myo19 KO cells. However, these data take into account a fixed number of cells, not a fixed amount of mitochondria. While this might indicate less mitochondrial mass per cell, it could also mean cells are smaller. In fact, Figure 5F appears to support the idea that mitochondrial content in the cell is unchanged, as VDAC1 and the complex components do not change when normalised to actin or GAPDH. The mitochondria-centric interpretation again does not consider the possibility that Myo19 might act on other cellular structures.

© 2021. Published by The Company of Biologists under the terms of the Creative Commons Attribution License (https://creativecommons.org/licenses/by/4.0/). 

Mitochondrial mass based on western blots appears to be comparable between WT and Myo19 ko cells. However, in flow cytometry a small fraction of Myo19 KO cells contained a slightly reduced mitochondrial mass. But cell size was not altered in Myo19 KO cells as monitored by flow cytometry. Since the seahorse data refer specifically to mitochondrial activity and were collected in Myo19-deficient cells that contain neither a mitochondrial nor a cytosolic pool of Myo19, it can be excluded that Myo19 acts on other cellular structures.

4. The lower number of focal adhesions (FA) is interpreted in a mitochondria-centric way as the result of decreased ATP production. If true, this decrease in ATP should be measurable, any condition causing the same reduction in ATP should yield the same FA phenotypes, and most importantly these phenotypes should be rescuable by tinkering with the energy status of the cell to increase ATP (or with extracellular ATP https://jcs.biologists.org/content/132/7/jcs223925, or phosphocreatine?). Another possibility is that a non-mitochondrial pool of Myo19 accounts for the phenotypes observed. After all, actin dynamics are required for focal adhesion and heavily influenced by myosins. So this whole part is too preliminary to be conclusive.

While it is true that we do not know the molecular mechanism by which Myo19 regulates focal adhesions, there is ample evidence for a cross-talk between mitochondria and focal adhesions, e.g. the loss of the OMM protein Miro1 caused a similar phenotype. Tinkering with ATP levels might itself induce complex (and non physiological) cellular responses that will be very difficult to interpret. Indeed, an increase of extracellular ATP led to overall changes in cell morphology, prohibiting a meaningful analysis. However, we newly included data showing that quenching of increased ROS levels normalizes focal adhesions.

5.The authors state “that the loss of Myo19 is not affecting ER morphology and the regulation of mitochondrial fusion/fission by Myo19 is not ER-dependent”. There is insufficient evidence to support this claim. Firstly, the staining presented would make it very difficult to ascertain if there were any morphological changes in the ER. More importantly, however, the role of the ER in mitochondrial fission is elicited through ER-mitochondria contact sites, which the authors do not study.

We fully agree with this point and thank the reviewer for pointing this out. We have omitted the statement that Myo19 is not affecting mitochondrial fission through ER-mitochondria contacts.

Minor comments:
1.Some of the image labels in Figure 4I are not readable.

We have corrected the image labels according to JCS guidelines.

2.What is “directionality” defined as with regards to peroxisomal motility?
We have removed the data on peroxisomal motility as it did not fit to the main thrust of the manuscript and requires further investigation.

Reviewer 2 Comments for the Author:

My main concerns are:
1. Overall, this manuscript contains a large body of work and detailed quantifications of phenotypes, however, it is a list of findings and fails to connect the separate observations into one story furthering our understanding on the cellular functions of MYO19. I would recommend to remove the last figure and extend the findings on the impact of MYO19 depletion on peroxisome motility in a separate manuscript.

We thank the reviewer for this suggestion. We have removed the data on peroxisome motility as it is not part of the main thrust of the manuscript and requires further investigations.

2. Unfortunately, some of the main findings, in particular the cell division defect and the role of MYO19 for mitochondria distribution during mitosis, has already been shown by Rohn et al. 2014.

© 2021. Published by The Company of Biologists under the terms of the Creative Commons Attribution License (https://creativecommons.org/licenses/by/4.0/).

We were able to confirm the findings of Rohn et al. (2014) using a different experimental set-up. Whereas they ablated Myo19 using an acute partial knockdown in HeLa cells, we used a stable deletion of Myo19 in HEK293 cells. Even more importantly, we significantly extended the findings of Rohn et al. (2014). We show that at prometaphase mitochondrial fusion is blocked in WT cells, but not in Myo19 ko cells. Myo19 regulates at prometaphase the mitochondrial association of adaptors for microtubule-based motors and the fission protein Drp1. Furthermore, we show that at mitosis the activities of the entire Myo19 protein are necessary to prevent the lack of mitochondrial fragmentation and the asymmetric distribution to the spindle poles. In addition, the loss of Myo19 causes a slower cell proliferation, reduced OCR, increased levels of ROS and altered focal adhesions in HEK293 cells. Reduced focal adhesion numbers and altered focal adhesion shape are rescued by the Myo19 tail and quenching of increased ROS levels.

3. Although it is very recommendable that the authors have made great efforts to quantify their observations, the statistical analysis is either completely missing for crucial experiments or lacking detail (is it the mean ±SEM that has been plotted or mean ± SD?). In addition, for multiple comparisons as is often the case in this manuscript, a Student's t-test or Mann-Whitney test are not appropriate and instead an ANOVA with post-hoc test, such as Bonferroni, should be used. Most importantly, 'n' does not represent the number of cells analysed in each experiment, but is the number of independent repeats of each experiment. This is particularly confusing as, for example, in Figure 4B-E four points are plotted but the n number is stated as 90.

We have added information to statistical methods that were used in the Materials and Methods section. We state now number of cells (N), number of independent experiments (n) and other relevant parameters. Bar graphs show mean values with ±SEM. One-way ANOVA with post-hoc Bonferroni test was additionally performed for multiple comparisons.

In figure 1A the MYO19 KO cells were analysed by immunoblotting, which shows still a band at the size of MYO19 in the KO cells. Is this still a mixed clone of KO and WT cells?

This is an unspecific band running slightly below Myo19 that is detected by the abcam antibody that was used for this blot. This band was not observed with the Sigma antibody. But this antibody is no longer available.

The results in figure 1 C, C', D and D' describe that absence of MYO19 leads to division failure causing an increase in multinucleated cells. The importance of MYO19 for cell division has already been shown in Rohn et al., 2014. The same paper also reported previously that in MYO19 siRNA KD cells mitochondria are asymmetrically distributed and accumulate inappropriately at spindle poles during anaphase, which are very similar to the results shown in figure 1 E, F, G and H.

For the answer see above as this point is identical to a previous comment (2.). We first confirm and then extend the findings of Rohn et al. as detailed above. We fully describe and acknowledge the findings of Rohn et al. in our manuscript.

In figure 2 the authors perform rescue experiments using wildtype and mutant forms of MYO19 and show that the phenotypes described in figure 1 and by Rohn et al. can only be rescued by fully functional MYO19. This is a valuable control experiment, but does not give any new insights into the cellular functions for MYO19.

Thank you for acknowledging that these are valuable experiments. They also show that it is not simply the interaction of the Myo19 motor domain with F-actin that is responsible for the phenotype as was suggested by the work of Rohn et al.

The results in figure 3 show that MYO19 remains associated with mitochondria during mitosis (figure 3 A-C) and that in MYO19 KO cells slightly increased levels of dynactin and TRAK1 are present on mitochondria. The authors then use two different approaches to inhibit dynein activity by either using the drug Dynarrestin or by depleting a subunit of the dynactin complex. Depletion of the dynactin subunit is able to prevent polewards movement of mitochondria, suggesting that dynein activity is inhibited and required for mitochondrial transport towards the minus end of microtubules at the spindle poles. Dynarrestin, however, leads to an increase in localisation of mitochondria at spindle poles in wildtype cells? This requires an explanation.

© 2021. Published by The Company of Biologists under the terms of the Creative Commons Attribution License (https://creativecommons.org/licenses/by/4.0/).

Kinesin- and dynein-dependent movements of mitochondria are coupled and the two independent modes of dynein inhibition might interfere differently with this coupling. Furthermore, microtubules pointing in all directions might differently affect directed movement of bridging mitochondria based on the mode of inhibition and altered coupling of the two motors. We added in the Discussion that indirectly also kinesin activity might be affected.

In figure 4 the authors present results on the impact on MYO19 KO on mitochondria morphology. The authors describe that in MYO19 KO cells “the number of mitochondrial individuals and networks was decreased” and “additionally a highly interconnected network with more branching was revealed”. This seems to be very contradictory. Please explain.

A smaller number of individual mitochondria and individual networks is consistent with few more extended and interconnected mitochondrial networks.

In an attempt to explain a link between MYO19 KO and changes in mitochondria morphology, the authors perform cellular fractionation experiments and identified a very slight increase in DRP1 levels associated with mitochondria in MYO19 KO cells; the change in DRP1 levels as shown on the blot is not convincing. Thus, it is crucial to include the correct statistical analysis and p-value in figure 4 L.

We fully agree and that is what we have done. The p-value was added to Fig. 4K. Note that the analysis is based on several independent blots.

In the next figure the authors switch to investigate mitochondrial function not in mitotic cells but during interphase, which is not linked to the experiments performed in figure 1-4. The authors identify changes in mitochondrial mass between WT and KO cells by flow cytometry using two different dyes, however, the differences were dismissed as no changes in protein levels of the various OXPHOS complexes were observed. Why can the flow cytometry measurements be ignored?

The flow cytometry measurements are not ignored, but they are indicating mitochondrial mass per cell whereas the western blots are relating mitochondrial protein levels to total protein. Moreover it is also acknowledged that changes in the Mitotracker mean fluorescence observed by flow cytometry may be due to enhanced ROS generation as oxidative stress was shown to affect it (Doherty and Perl, 2017).
Doherty, E. and Perl, A. (2017) ‘Measurement of Mitochondrial Mass by Flow Cytometry during Oxidative Stress’, Reactive Oxygen Species, 4(10), pp. 275–283. doi: 10.20455/ros.2017.839.

In the next set of experiments the authors change focus again and demonstrate the loss of MYO19 causes a reduction in cell adhesion. No obvious link between MYO19 - mitochondria and focal adhesions was observed, only a small change in expression levels of focal adhesion associated proteins such as vinculin and paxillin.

In the Discussion we refer to multiple independent studies that have reported a cross-talk between mitochondria and focal adhesions. We included new data that demonstrate a link between focal adhesions and ROS generation. An increase in the number, size and length of focal adhesions was observed upon ROS scavenger treatment in cells lacking Myo19.

In the final figure the authors describe a change in peroxisome velocity in MYO19 KO cells, which is an interesting observation, since MYO19 cannot be localised to peroxisomes. However, this set of experiments again is a stand-alone observation, which is not linked to either the role of MYO19 during mitochondria distribution during mitosis, for regulating mitochondria activity or for a potential link of MYO19 to focal adhesions.

We have removed the stand-alone observation (final figure) concerning peroxisomes.

Further points:

The title is not capturing the focus of the manuscript, since microtubule dynamics is not analysed in interphase cells and no insight in the role of microtubule motors is provided.

© 2021. Published by The Company of Biologists under the terms of the Creative Commons Attribution License (https://creativecommons.org/licenses/by/4.0/).

We have changed and shortened the title to capture better the content of the described work.

An overview on the observed phenotype and proposed roles of MYO19 would be very helpful.

A summary table of the observed phenotype and proposed roles of Myo19 was added to the manuscript.

Reviewer 3

Specific comments:

1: Because Myo19 KO resulted in a 40% decrease in ATP production, it seems possible that some of the changes the authors report are due to the decreased ATP levels or changed ATP/ADP ratios that would secondarily affect the activity of other motor proteins and/or kinases. The authors should mention this possibility in their discussion, especially given the relatively modest effects of Myo19 KO on peroxisome motility.

Thank you for pointing out this possibility. We have added in the Discussion that altered ATP levels or ATP/ADP ratios could contribute to the observed phenotypes. In this context we would like to point out that we newly show that increased ROS levels play an important role in reducing cell-matrix adhesion. The peroxisome data were removed as suggested by others.

2: The paper by Bocanegra et al (2019) reporting Miro-dependent and Miro-independent mechanisms of Myo19 interaction with mitochondrial membranes should be cited in the introduction or discussion. Given that KO of the actin-based motor Myo19 perturbs the distribution of mitochondria during mitosis, the authors should also consider citing the recent MBOC paper by AM Kita et al that demonstrates the presence of F-actin in vertebrate spindles.

We have added a reference to the Bocanegra et al. (2019) paper. We have decided against citing the paper by AM Kita et al., because we do not see any direct relation to our work.

3: To unambiguously specify the constructs used here whose sequences have not already been published or deposited in Addgene, the authors should include the full nucleotide sequences of the constructs they generated in the supplemental material. This would also enhance reproducibility by providing a permanent record of the exact vector and insert sequences, along with any linker sequences, splice forms, or sequence variants.

We believe that the construction of the plasmids is adequately described in the Materials and Methods section and Suppl. Fig. S2. We will be happy to provide the plasmids and further information upon request.

Minor comments:

70: Add MEF to the list of abbreviations.

MEF and additional abbreviations were added to the list of abbreviations.

106: CRIPR should be CRISPR

This was corrected.

109: Did the phenotype of the CRISPR Myo19-KO cells weaken with the number of passages? If so, this should be mentioned, and the range of passages used for the experiments should be stated.

We observed no weakening of the phenotypes, but we avoided higher passage numbers. Cells were analysed at passages 3 to 20.

120: The authors should clarify what they mean by “struggled to divide”.

© 2021. Published by The Company of Biologists under the terms of the Creative Commons Attribution License (https://creativecommons.org/licenses/by/4.0/).

It seemed that they made several attempts to divide and it took longer for them to divide. We have clarified this.

126: For each mean reported here and in the rest of the manuscript, the authors should include the standard deviation or SEM.

The SEM was newly included where it was missing (Figs. 1F-H; 2F-H; 3 B,C, F-K; 4B-E, G; 6 D).

140: Is the G137R mutation expected to result in a mutant that cannot bind to any nucleotide and is trapped in rigor? This should be mentioned and could result in an unnatural gain-of-function phenotype where mitochondria are anchored to actin.

An explanation of the predicted consequences for Myo19 motor function by this point mutation is included. We did not observe any alterations in the actin organization associated with mitochondria or the association of mitochondria with F-actin. We have added this information.

199: This sentence is not clear.

We have rephrased this sentence.

267-268: The last part of this sentence needs to be changed. The authors show that overall ER morphology is independent of Myo19, but they do not show that Myo19 regulation of mitochondrial fusion/fission is independent of ER this would require perturbing or eliminating the ER.

We have deleted this statement.

281: It would be more accurate to state that no differences in phosphorylation were detected.

We have changed this as suggested.

306: Add FCCP to the list of abbreviations and provide more background on the experiments in Figure 5H-I and the use of oligomycin, FCCP, and rotenone.

We have added FCCP to the list of abbreviations and provide more background for the use of oligomycin, FCCP, and rotenone.

356: The authors should specify if the mean velocity reported here corresponds to the net displacement divided by the 2 minute recording time, the mean of ~100 nm displacements recorded once per second, or something else. Because velocity is a vector quantity and speed is scalar, it is important to know how movements in opposite directions were dealt with when calculating the mean.

We have removed the peroxisome data as suggested by reviewer 2.

538: Zhang et al., 2014 is missing from the references.

We have replaced the Zhang et al. by the Cong et al. reference and added it to the list of References. Cong L, Ran FA, Cox D, Lin S, Barretto R, Habib N, Hsu PD, Wu X, Jiang W, Marraffini LA, Zhang F. (2013) Multiplex genome engineering using CRISPR/Cas systems. Science. 339(6121):819-23.

661: State whether the image analyses and measurements were performed blinded.

The number of cell nuclei was analysed blinded.

© 2021. Published by The Company of Biologists under the terms of the Creative Commons Attribution License (https://creativecommons.org/licenses/by/4.0/).

Second decision letter

MS ID#: JOCES/2020/255844

MS TITLE: Coordination of mitochondrial and cellular dynamics by the actin-based motor Myo19

AUTHORS: Katarzyna Majstrowicz, Ulrike Honnert, Petra Nikolaus, Vera Schwarz, Stefanie Jennifer J Oeding, Sandra A Hemkemeyer, and Martin Baehler
ARTICLE TYPE: Research Article

We have now reached a decision on the above manuscript.

To see the reviewers' reports and a copy of this decision letter, please go to: https://submit-jcs.biologists.org and click on the 'Manuscripts with Decisions' queue in the Author Area. (Corresponding author only has access to reviews.)

As you will see, the reviewers gave favourable reports but raised some minor points that will require amendments to your manuscript. I hope that you will be able to carry these out, because I would like to be able to accept your paper.

We are aware that you may be experiencing disruption to the normal running of your lab that makes experimental revisions challenging. If it would be helpful, we encourage you to contact us to discuss your revision in greater detail. Please send us a point-by-point response indicating where you are able to address concerns raised (either experimentally or by changes to the text) and where you will not be able to do so within the normal timeframe of a revision. We will then provide further guidance. Please also note that we are happy to extend revision timeframes as necessary.

Please ensure that you clearly highlight all changes made in the revised manuscript. Please avoid using 'Tracked changes' in Word files as these are lost in PDF conversion.

I should be grateful if you would also provide a point-by-point response detailing how you have dealt with the points raised by the reviewers in the 'Response to Reviewers' box. Please attend to all of the reviewers' comments. If you do not agree with any of their criticisms or suggestions please explain clearly why this is so.

Reviewer 1

*Advance summary and potential significance to field*

The authors have added further support of several aspects of their work. For example, the work showing that treatment with the ROS scavenger MPG rescues the number of focal adhesions per cell in the Myo19-KO cells is particularly convincing to causally link focal adhesions to mitochondria.
It is still unclear why the authors are so confident that all phenotypes observed are solely driven by Myo19 on the mitochondria given that: a) Myo19 artificially targeted to the mitochondria does not rescue mitochondrial asymmetry in Myo19 KO cells, nor any non-mitochondrial phenotype; b) Lopez-Domenech et al. (2018) shows that a cytoplasmic pool of Myo19 exists in wild-type cells; c) the very Figure 3D and 4I of this manuscript both show a cytoplasmic pool of Myo19 in wild-type cells; d) the fact that the pool of Myo19 that resides in the cytoplasm is less stable than that on the mitochondria does not preclude Myo19 having functions outside of the mitochondria (as argued in the rebuttal letter). e) the ER asymmetrically segregates during mitosis in Myo19 KO cells.
This ER asymmetry in particular is worrisome, as it strongly suggests a non-mitochondrial role for Myo19. Such non-mitochondrial role of Myo19 could therefore be behind several of the phenotypes herein, in particular the failed mitosis and binucleation observed. The authors state in their rebuttal that ”observed phenomena can be traced back to mitochondrial alterations”. While they CAN be in theory traced back, the burden of the proof that they indeed trace back to mitochondria remains with the authors. This proof is not established as artificially targeting Myo19 to mitochondria does not work.

© 2021. Published by The Company of Biologists under the terms of the Creative Commons Attribution License (https://creativecommons.org/licenses/by/4.0/). 

In the case of focal adhesion, as stated above, the ROS scavenging makes a good case to trace the phenotype back to mitochondria.
Concerning the ER asymmetry, the topic is left unexplored by the authors. Fortunately for them, recently published work (DOI: 10.1038/s41586-021-03309-5) might aid in tracing the Myo19-dependent ER asymmetry observed back to mitochondria.

*Comments for the author*

In any case, a thorough acknowledgement of a possible non-mitochondrial role for Myo19 should be discussed.
Other points
-The authors claim that the fact that Dynein inhibition affects mitochondrial distribution in mitosis "is contrary to the reported dissociation of microtubule-based motors from mitochondria at mitosis (Chung, Steen and Schwarz, 2016).". It is not really. In fact the effect of Dynein inhibition on mitochondria may not necessarily involve its association with mitochondria", but could be wrought be completely independent mechanisms (e.g. cytoplasmic streaming).
- "This finding implies a role for Myo19 and mitochondria in ER, but not peroxisome, organisation during late stages of mitosis and cell division.". This language is very strong for subtle differences in p-values. A better sentence would be "This finding implies a role for Myo19 and mitochondria in ER organisation during late stages of mitosis and cell division. No effect on peroxisome organisation could be detected".

Other minor comments
1. How are the "mitochondrial networks" defined in Figure 4?
2. Define ELM

Reviewer 2

*Advance summary and potential significance to field*

As described in my first review of the manuscript. The is an assessment of the revised manuscript.

*Comments for the author*

Overall the authors have done a good job revising the manuscript, which is greatly improved.

The statistical analysis, however, is still unclear in places. In the legend for figure 1 F-G the number (n) of independent experiments is still missing and it is not clear which of the two statistical methods mentioned in the legend were used for the analysis.

In Figure 3E and 4 J, K it should be stated whether a paired or unpaired t-test was used. Often the number of cells but not the experimental repeats that were used for stats test are included. Normally it is stated the other way around to give confidence in the data e.g. n=3 with >60 cells analysed.

Reviewer 3

*Advance summary and potential significance to field*

The revisions to this manuscript strengthen it and satisfactorily address my concerns from the initial review. The removal of the peroxisome motility data both strengthens and simplifies the paper. By using CRISPR mediated KO of Myo19 in HEK cells, the authors definitively confirm the core result from Rohn et al (obtained by RNAi mediated knockdown in HeLa cells) that loss of Myo19 results in asymmetric distribution of mitochondria during mitosis. The current manuscript extends this work with a detailed analysis of the cellular phenotype of Myo19 KO cells, with the new table summarizing the the Myo19 KO phenotype being particularly useful. A key novel result is the surprising finding that targeting the Myo19 motor domain to mitochondria is not sufficient for rescue, demonstrating that the tail (which binds Miro) is also required. Most importantly, this

© 2021. Published by The Company of Biologists under the terms of the Creative Commons Attribution License (https://creativecommons.org/licenses/by/4.0/). 

manuscript is the first to report that loss of Myo19 results in dramatic defects in mitochondrial function as indicated by results such as a ~40% decrease in ATP production.

*Comments for the author*

A minor comment the authors may wish to deal with is that on line 121-122 Adikes et al is cited following the statement that the 3 IQ motifs bind to regulatory light chains, but Adikes used co-expression in insect cells to show that Myo19 can bind to 3 calmodulin light chains per heavy chain, while Lu et al provide evidence that the 3 IQs in Myo19 preferentially bind to Regulatory Light Chains.

---

**Second revision**

Author response to reviewers' comments

**Point-by-point response:**

**Reviewer 1** Comments for the Author:
1. In any case, a thorough acknowledgement of a possible non-mitochondrial role for Myo19 should be discussed.
We newly acknowledge in the Discussion that a non-mitochondrial cytosolic pool of Myo19 could be involved in the observed phenotypes and we have toned down the role of the tail region in coordinating microtubule-dependent force production.

2. The authors claim that the fact that Dynein inhibition affects mitochondrial distribution in mitosis "is contrary to the reported dissociation of microtubule-based motors from mitochondria at mitosis (Chung, Steen and Schwarz, 2016).". It is not really. In fact the effect of Dynein inhibition on mitochondria may not necessarily involve its association with mitochondria", but could be wrought be completely independent mechanisms (e.g. cytoplasmic streaming).
This statement was removed.

3. "This finding implies a role for Myo19 and mitochondria in ER, but not peroxisome, organisation during late stages of mitosis and cell division.". This language is very strong for subtle differences in p-values. A better sentence would be "This finding implies a role for Myo19 and mitochondria in ER organisation during late stages of mitosis and cell division. No effect on peroxisome organisation could be detected".
We have replaced this sentence with the more accurate sentence that was suggested by the reviewer.

Other minor comments
1. How are the "mitochondrial networks" defined in Figure 4?

Mitochondrial networks and all other parameters in Figure 4 are defined as described in Valente, A. J. *et al*. (2017) 'A simple ImageJ macro tool for analyzing mitochondrial network morphology in mammalian cell culture', *Acta Histochemica*, 119(3), pp. 315-326. doi: 10.1016/j.acthis.2017.03.001. The analysis was performed with the semi-automated MiNA toolset described in this publication. This is mentioned in Materials and Methods section.
'Networks' are the number of objects in the image that contain at least 1 junction pixel and are thus comprised of more than one branch.

2. Define ELM

We refer to each Miro EF hand pair with ligand mimic helix arrangement as an ELM domain. We defined the term in the text of the Introduction and added this abbreviation and its definition to the list of abbreviations.

© 2021. Published by The Company of Biologists under the terms of the Creative Commons Attribution License (https://creativecommons.org/licenses/by/4.0/).

**Reviewer 2** Comments for the Author:

1. The statistical analysis, however, is still unclear in places. In the legend for figure 1 F-G the number (n) of independent experiments is still missing and it is not clear which of the two statistical methods mentioned in the legend were used for the analysis.

In Figure 3E and 4 J, K it should be stated whether a paired or unpaired t-test was used. Often the number of cells but not the experimental repeats that were used for stats test are included. Normally it is stated the other way around to give confidence in the data e.g. n=3 with >60 cells analysed.

We have added to the legend of figures 1 F-H the number (n) of independent experiments that were performed and clarified which statistical method (ANOVA with Bonferroni post-hoc test) was used.
To the legends of Figure 3E and 4 J, K we have added the information that a paired student t-test was used. We have modified the legends where necessary, so that we conform to first mentioning the number of independent experiments followed by the number of cells analysed.
Number of experimental repeats was added to each figure legend where it was missing.

**Reviewer 3** Comments for the Author:

A minor comment the authors may wish to deal with is that on line 121-122 Adikes et al is cited following the statement that the 3 IQ motifs bind to regulatory light chains, but Adikes used co-expression in insect cells to show that Myo19 can bind to 3 calmodulin light chains per heavy chain, while Lu et al provide evidence that the 3 IQs in Myo19 preferentially bind to Regulatory Light Chains.

We have moved the Adikes et al reference to a more appropriate place and refer now specifically to Lu et al. for showing that the IQ motifs of Myo19 bind to regulatory light chains.

---

Third decision letter

MS ID#: JOCES/2020/255844

MS TITLE: Coordination of mitochondrial and cellular dynamics by the actin-based motor Myo19

AUTHORS: Katarzyna Majstrowicz, Ulrike Honnert, Petra Nikolaus, Vera Schwarz, Stefanie Jennifer J Oeding, Sandra A Hemkemeyer, and Martin Baehler
ARTICLE TYPE: Research Article

I am happy to tell you that your manuscript has been accepted for publication in Journal of Cell Science, pending standard ethics checks.

© 2021. Published by The Company of Biologists under the terms of the Creative Commons Attribution License (https://creativecommons.org/licenses/by/4.0/).
